# Extracellular vesicles: a rising star for therapeutics and drug delivery

**DOI:** 10.1186/s12951-023-01973-5

**Published:** 2023-07-20

**Authors:** Shuang Du, Yucheng Guan, Aihua Xie, Zhao Yan, Sijia Gao, Weirong Li, Lang Rao, Xiaojia Chen, Tongkai Chen

**Affiliations:** 1 Science and Technology Innovation Center, Guangzhou University of Chinese Medicine, 12 Jichang Road, Guangzhou, 510405 China; 2 State Key Laboratory of Quality Research in Chinese Medicine, Institute of Chinese Medical Sciences, University of Macau, Room 6007, N22, Taipa, 999078 Macau SAR China; 3 Institute of Biomedical Health Technology and Engineering, Shenzhen Bay Laboratory, Shenzhen, 518132 China

**Keywords:** Extracellular vesicles, Drug loading, Surface modification, Targeted therapy, Neurodegenerative diseases, Clinical challenges

## Abstract

Extracellular vesicles (EVs) are nano-sized, natural, cell-derived vesicles that contain the same nucleic acids, proteins, and lipids as their source cells. Thus, they can serve as natural carriers for therapeutic agents and drugs, and have many advantages over conventional nanocarriers, including their low immunogenicity, good biocompatibility, natural blood**–**brain barrier penetration, and capacity for gene delivery. This review first introduces the classification of EVs and then discusses several currently popular methods for isolating and purifying EVs, EVs-mediated drug delivery, and the functionalization of EVs as carriers. Thereby, it provides new avenues for the development of EVs-based therapeutic strategies in different fields of medicine. Finally, it highlights some challenges and future perspectives with regard to the clinical application of EVs.

**Graphical Abstract:**

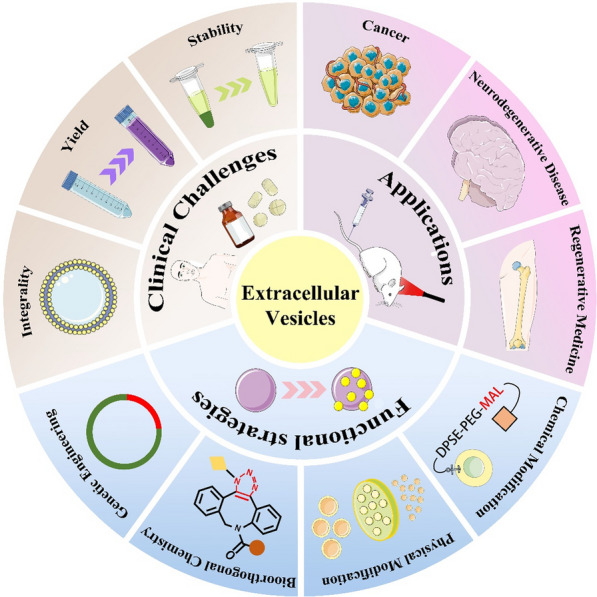

## Highlights

- Various current techniques for isolating extracellular vesicles are reviewed, and their advantages and disadvantages are compared.
- An overview of the strategies used for the modification of extracellular vesicles and their application as delivery systems or therapeutic agents in different diseases is provided.
- Several challenges in the clinical application of extracellular vesicles-based nanoplatforms are discussed, along with solutions for their implementation as a promising therapeutic tool.

## Introduction

Extracellular vesicles (EVs) are nanosized lipid bilayer vesicles that are actively secreted by cells and can be derived from a wide range of sources. They have been detected in biological samples and cell cultures obtained from human patients; in cells of non-human origin (e.g., cells of animal origin); and in plants, bacteria, fungi, parasites, and other species and have been applied in research [1]. EVs carry many active biomolecules, including nucleic acids, proteins, lipids, and carbohydrates, as internal cargo and surface-associated molecules [2]. They transport these components from the donor cell to the recipient cell via various mechanisms, such as direct membrane fusion, receptor–ligand interaction, endocytosis, and phagocytosis [3]. The secretion of EVs was initially thought to be responsible for removing unwanted substances from cells. However, studies later showed that EVs are involved in a variety of intercellular signaling pathways, mediating various physiological and pathological cellular processes by transporting different biomolecules and achieving intercellular component exchange [4]. Considering that EVs can deliver bioactive molecules and cross biological barriers, they are increasingly being explored as potential therapeutic agents [5].

EVs act as carriers and can deliver cargo to specific intracellular locations in a target-specific manner via the plasma membrane [6]. Unlike conventional nanocarriers, EVs are cell-derived and therefore have low immunogenicity and toxicity [7]. After being wrapped with unique biomolecules, EVs are endocytosed by receptors on target cells. They deliver their cargo and convey genetic information, protecting the cargo from degradation and crossing biological barriers (e.g., blood–brain barrier [BBB]) during the delivery process [8, 9]. They also improve the half-life of the cargo, have better biocompatibility, and serve as a safe vehicle for drug delivery [7]. EVs have been applied in the treatment of cancer [10], neurodegenerative diseases [11], and regenerative medicine [12], among other conditions. Primarily, there are two different use cases for EVs. In the first case, the natural biological function of EVs is leveraged to target the tissue of interest and reduce pathological signals, or to mimic the natural reparative process. In the other case, EVs are used as carriers to deliver therapeutic agents to target sites [13]. Since EVs were reported to have applications as carriers of anti-inflammatory drugs [14], more attention has been paid to EVs-mediated drug delivery systems. In addition, there is a rich availability of EVs sources. In vitro, the most commonly used cell sources of EVs for drug delivery are immune cells, mesenchymal stem cells (MSCs), cancer cells, and frequently used commercial cell lines (e.g., HEK293T) [15]. In vivo, EVs are present in various biological fluids, such as blood, urine, saliva, and ascites [16]. This suggests that EVs could serve as a desirable platform for biomedical applications. It has been reported that EVs can be applied as platforms for liquid biopsies [17]. Since EVs are present in some bodily fluids, they can capture cargo from dysfunctional cells and serve as a new source of biomarkers for liquid biopsies as well as therapeutic targets [18]. This demonstrates the importance of EVs in disease diagnosis and treatment.

This paper reviews the recent advances in our understanding of the processes involved in EVs secretion, related isolation and purification techniques, the use of EVs as therapeutic agents or nanocarriers along with modifications, and their therapeutic applications, primarily focusing on EVs of animal origin. Thus, it provides improved insights into the current status and future directions of research in this field.

## Classification of extracellular vesicles

**Fig. 1:**
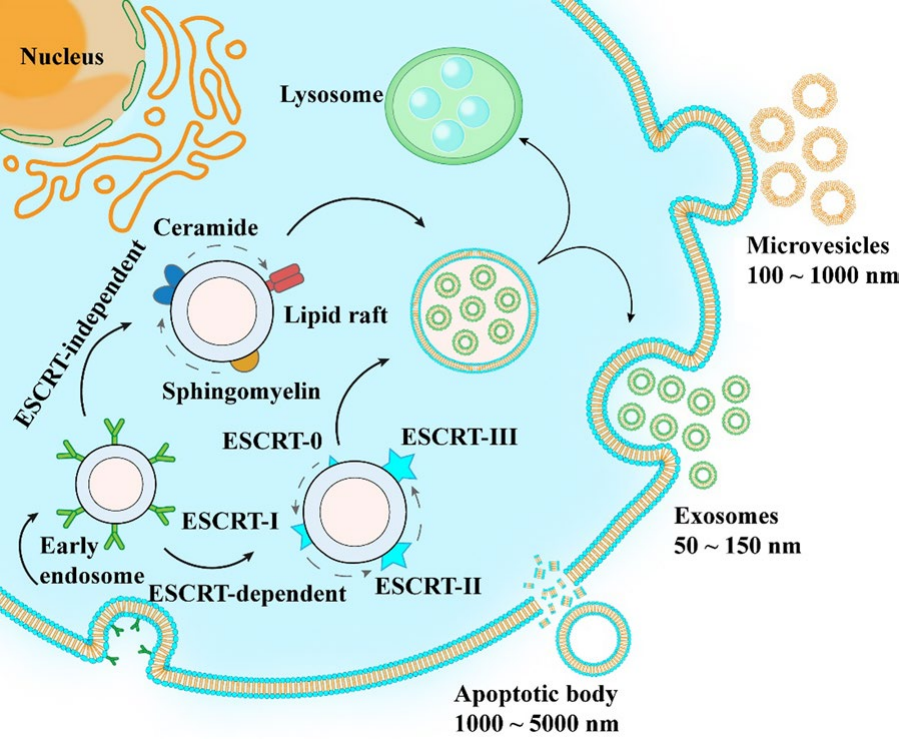
Formation and release of exosomes, microvesicles, and apoptotic vesicles

EVs is a term used to describe a population of heterogeneous vesicles ranging from 40 to 1000 nm in size, encapsulated by lipid bilayers, and released by various cell types [19]. In fact, there are several subtypes of EVs, and this classification is based on their size, biogenesis, and the expression or absence of specific proteins [20]. EVs can be broadly classified into three groups: exosomes, microvesicles, and apoptotic vesicles. The process of their formation is illustrated in Fig. 1. With progress in research on EVs, other types of EVs, such as ectosomes, microparticles, and oncosomes have also been identified [21]. Since the contents of different EVs are highly heterogeneous depending on the recipient or source cells and the biogenesis of their subtypes is difficult to elucidate [22], we focused on the three broader categories mentioned above (exosomes, microvesicles, and apoptotic vesicles). The differences between the three main types of EVs are summarized in Table 1.

**Table 1:** Main types of extracellular vesicles and the differences among them

Type and size (nm)	Origin	Biogenesis	Density (g/m)		Appearance	Major pathway	Biomarkers		Contents	Refs.
Exosome 50–150	Endosomal membrane	The fusion of multivesicular bodies and plasma membranes	1.13–1.18		Cup-shaped	ESCRT-dependent	Tetraspanins (CD9, CD81, CD63, CD82) Alix TSG101 HSP70 ESCRT		Nucleic acids (mRNA, miRNA, Pre-miRNA, snRNA, mtDNA, dsDNA, etc.) Lipids Proteins (cytoskeletal, heat shock, nuclear enzyme, etc.) Amino acids Metabolites MHC	[28, 40, 232, 235]
Microvesicles 100–1000	Plasma membrane	Shedding from the plasma membrane	1.04–1.07	Cup-shaped		Ca^2+^-dependent		CD40 Integrin Selectin	Nucleic acids (miRNAs, etc.) Lipids Membrane protein enzymes Growth factor- receptors, Cytokines Chemokines	[33, 34, 39, 40, 232, 236]
Apoptotic bodies 1000–5000	Plasma membrane, Endoplasmic reticulum	Direct outward budding of the cell membrane in dying cells	1.16–1.28	Heterogeneous		Apoptosis-related pathway		Histone proteins, Annexin V Thrombospondin C3b	Nucleic acids (mRNA, miRNA, fragments of DNA), Lipids Cell organelles	[33, 34, 40, 232, 236]

Notably, diverse culture conditions, methods of isolation, and purification protocols may result in the formation of different subpopulations. Further, the overlap between vesicle size and the lack of specific biogenetic markers for the identification of EVs subtypes have led to conflicting definitions in the literature [23]. Therefore, the International Society for Extracellular Vesicles (ISEV) recommends the use of “extracellular vesicles” as the universal fate method for defining the cellular release of vesicles [22].

### Exosomes

Exosomes are relatively small extracellular vesicles, ranging in size from approximately 50–150 nm in diameter [24]. Their biogenesis occurs via the endocytic endosomal pathway, in which the cytoplasmic membrane buds inward, leading to the capture of membrane molecules and the formation of early endosomes within cells [19, 25]. During subsequent maturation, early endosomes fuse to form late endosomes, resulting in the invagination of the endosomal membrane into the lumen to form intracellular vesicles (ILVs), which in turn form multivesicular bodies (MVBs) [26, 27]. MVBs may fuse with lysosomes, which results in their degradation, or fuse with the plasma membrane and subsequently release exosomes into the extracellular compartment as cytosolic vesicles [22, 28, 29]. Studies have demonstrated that MVBs formation is mediated by two different pathways. The first pathway is associated with the ESCRT endosomal sorting complex (ESCRT-0, -I, -II, and -III and Vps4 complexes) [30]. ESCRT-0 degrades ubiquitinated cargo, while ESCRT-I and ESCRT-II are responsible for the formation of endosomal membrane buds. ESCRT-III surrounds the neck of the formed vesicle, and Vps4 plays a role in the rupture of the membrane and finally the formation of the MVBs luminal vesicle [31, 32]. The second pathway does not rely on the ESCRT mechanism but is instead dependent on a lipid component of the endosomal membrane, which contains many sphingolipids. However, these sphingolipids represent substrates for neutral sphingomyelinase 2 (nSMase2). On the endosomal membrane, nSMase2 transforms sphingolipids into ceramides, subsequently inducing microdomains to merge into larger structures, leading to domain budding and ILVs formation [33–35]. This process involves the transport and signaling of many proteins, especially RAS-related proteins. In this process, Rab27A and Rab27B are key regulatory proteins, and Rab27A has also been found to be associated with the fusion of the MVBs with the plasma membrane [36].

### Microvesicles

Microvesicles (MVs) are 100–1000 nm in size, and unlike exosomes are formed by the detachment of the cell membrane after direct outward budding [37, 38]. The process of MVs shedding is associated with the molecular reassignment of the plasma membrane, which is in turn influenced by protein and lipid composition and Ca^2+^ levels [39]. The asymmetric distribution of phospholipids in the membrane maintains lipid “laterality”. Meanwhile, the inward flow of intracellular Ca^2+^ alters the asymmetric distribution of membrane phospholipids, wherein asymmetry is maintained by Ca^2+^-dependent enzymes. There is a switch to the outer layer and cleavage of cytoskeletal actin filaments, leading to the reorganization of the cytoskeleton and facilitating germination [40, 41]. Moreover, cytoskeletal elements as well as their regulators are essential for microvesicle biogenesis. Small GTPases from the RHO family and RHO-related protein kinases (ROCK) are important regulators of actin dynamics, and induce MVs biogenesis in different tumor cell populations [4, 42]. The small GTPases are classified as ADP-ribose factor (ARF), Rab22a, and Rho [43]. The ARF6 GTP/GDP cycle enables neck formation in tumor cells and allows MVs excision through extracellular signal-regulated kinase (ERK) recruitment and myosin light chain enzyme (MLCK) activation [42].

### Apoptotic bodies

Apoptotic vesicles are protrusions formed during programmed cell death via the bubbling of the apoptotic cell membrane, followed by disintegration. They have a particle size of 1000–5000 nm [44–46]. The membranes of apoptotic cells express markers that promote phagocytosis by macrophages or surrounding cells before rupture, thereby clearing apoptotic vesicles and regulating the immune system [47]. Phosphatidylserine (PS), for example, translocates from the inner lobe to the outer leaflet in the early stages of apoptosis and is believed to act as an “eat me” signal. In addition, PS may also serve as a tumor marker [48, 49]. However, most studies have focused on exosomes and microvesicles, and apoptotic vesicles have rarely been examined in the context of nanomedicine, likely owing to their size heterogeneity.

Meanwhile, in tumor cells, EVs can serve as signaling tools, regulating many cellular processes, including the promotion of cell growth, invasion [50], the stimulation of angiogenesis [51], and drug resistance [52]. In normal cells, they are involved in coagulation [53], regeneration [54], and immune regulation [55], among other processes. Since EVs have a complex composition with multiple physiological functions, their own intrinsic functions should be considered during their application as therapeutic vehicles [56]. EVs released into the tumor microenvironment (TME) or bodily fluids are taken up by receptor cells. The known pathways of EVs entry into cells are direct fusion with receptor cell membranes, receptor-mediated endocytosis, lipid raft interaction, reticulin interaction, and macrophage phagocytosis [57]. However, it has been reported that exosomes are mainly internalized by non-dependent lipid raft-mediated endocytosis and not via direct membrane fusion. Similar to other nanocarriers, internalized EVs can undergo endosomal escape and subsequently release the cargo into the cytosol. However, the acidic environment during endosomal escape and in the lysosomal pathway may lead to cargo degradation [58]. Overall, the mechanism of EVs uptake is complex and needs to be evaluated in greater depth.

## Extracellular vesicles isolation techniques

The small size, low density, and wide distribution of EVs in the complex bodily fluid environment make it quite challenging for researchers to obtain high-purity EVs following isolation and analysis. This also limits their clinical application [18]. Several new techniques and commercial products have now been developed to isolate EVs. These are based on separation principles that leverage the physical properties of EVs, such as their density, mass, and shape. In addition, separation can also be performed based on the physicochemical and biochemical properties of EVs, such as charge, hydrodynamics, solubility, and surface properties (proteins) [59]. Several conventional separation methods have been developed, each of which has its own advantages and disadvantages (Table 2). Accordingly, separation can be performed in a single or combined manner depending on the different properties of the EVs [60].

**Table 2:** Current techniques for the isolation of extracellular vesicles and their comparison

Isolation techniques (Mechanism of isolation)	Advantages	Disadvantages	Isolation time	Yield and purity	Refs.
Ultracentrifugation (Size; Density)	Simple; Cheap; Gold standard	Vulnerable to contamination by protein aggregates; Time-consuming; High demand for sample volumes; Expensive instruments; Low recovery rate; Structures easily damaged	> 4 h	Low yield and Low purity	[68, 237]
Density gradient ultracentrifugation (Size; Density)	High purity; Exosome subpopulations can be isolated	Time-consuming; Larger losses; Cumbersome operation	> 16 h	Low yield and High purity	[60, 68, 69]
Size exclusion chromatography (Size; Molecular weight)	Structural integrity; Low usage; Saves time and effort; Ability to isolate specific subgroups of EVs	Wider size distribution; Contaminants such as protein aggregates and lipoproteins; Special columns required	10–20 min per sample	High yield and High purity	[60, 238]
Ultrafiltration (Size; Molecular weight)	Simple; Low cost; Variable sample injection volume; Rapid	Easy to cause clogged pores; EVs are adsorbed on the filter surface; Leading to a loss of yield; Shear forces may damage EVs	~ 1 h for 200 mL cell culture media	High purity	[29, 239]
Field-flow fractionation (Size; Molecular weight)	Label-free; Gentle; Rapid; Highly reproducible; High resolution	Small sample capacity; Analytes need to be stratified and concentrated beforehand; Samples need to be graded according to sample size	< 1 h	High purity	[84, 240]
Precipitation-based methods (Solubility; Charge)	Easy to operate; Commercial kits are available; No specific equipment required	Protein aggregates may be precipitated; Commercial kits are expensive	0.3–12 h	High yield and Low purity	[68, 128, 241]
Microfluidics (Affinity; Density; Size; Acoustic; Electrophoretic)	Fast; Low sample consumption; High recovery rate; High yield; automation; High portability	High cost; need for external force; Not suitable for large-scale production and requires method validation; Sample may evaporate	< 1 h	High yield and High purity	[68, 128, 242]
Affinity-based methods (Affinity)	High specificity; Rapid	Cumbersome process; Long operation time; High cost; Not suitable for large-scale production; Low yield; Requires subsequent isolation and purification steps	4–20 h	Low yield and High purity	[68, 98]

### Ultracentrifugation

**Fig. 2:**
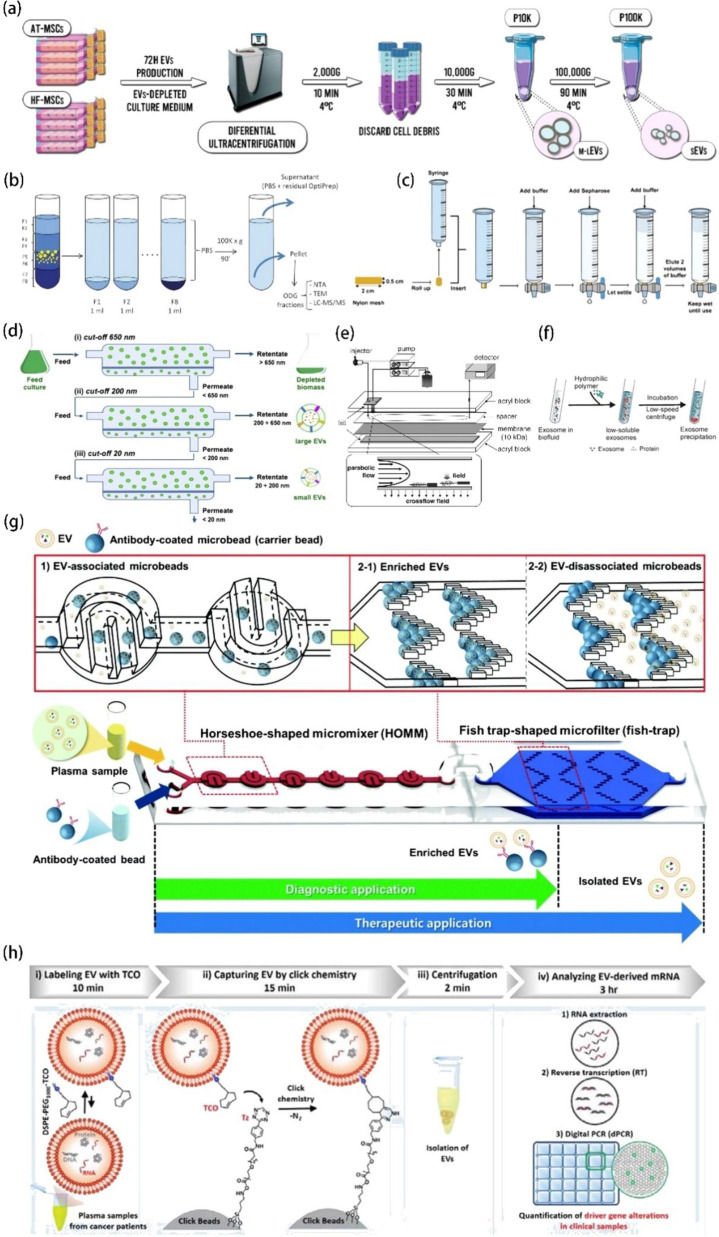
EVs isolation techniques. **a** Schematic showing the isolation method used for HF-EVs and AT-EVs. Reprinted with permission from Ref [63]. **b** EVs were collected in different fractions after Optiprep^™^ density gradient separation (ODG fractions). Reprinted with permission from Ref [70]. **c** Set-up of a size-exclusion chromatography (SEC) column using a syringe. Reprinted with permission from Ref [73]. **d** Schematic showing TFF steps and the retentate, permeate, and feed for the three filters used in sequence: (i) 650 nm, (ii) 200 nm, (iii) 20 nm (namely 500 kDa). Reprinted with permission from Ref [79]. **e** Configuration of the miniaturized frit-inlet asymmetrical flow field-flow fractionation (FI-AFlFFF) channel with an enlarged side view of the channel illustrating the parabolic flow velocity profiles and equilibrium positions of sample components experiencing two opposite forces (cross-flow field and diffusion). Reprinted with permission from Ref [87]. **f** Schematic of the polymer precipitation strategy. Reprinted with permission from Ref [60]. **g** Schematic illustration of the designed modular microfluidic chip. Reprinted with permission from Ref [98]. **h** Schematic illustration of a streamlined workflow for the capture and characterization of EVs. Reprinted with permission from Ref [100]

Ultracentrifugation (UC) is the most common method and the current gold standard of EVs separation [61]. In this method, EVs are separated because their settling coefficients differ from those of other particles [17]. Briefly, UC is a differential centrifugation method in which the centrifugal force is gradually increased, first removing dead cells and cell debris at a low centrifugal force of 2000–4000 ×*g*; removing apoptotic vesicles, MVs, biopolymers, etc., at 10,000–20,000 ×*g*; and precipitating exosomes at a high centrifugal force of 100,000–200,000 ×*g* [29, 60, 62]. Las Heras et al. reported the separation of hair follicle and adipose tissue mesenchymal stem cells -derived EVs using UC. In their study, cells containing culture medium were first collected and then centrifuged at 2000 ×*g* and 4 °C for 10 min to remove cell debris, and the supernatant was then centrifuged at 10,000 ×*g* for 30 min. Finally, this second supernatant was used to obtain EVs after centrifugation at 100,000 ×*g* for 90 min (Fig. 2a) [63]. Li et al. used UC to extract exosomes from prostate cancer cells and normal prostate epithelial cells, and they developed a 3D-SiO_2_ porous chip for mouse tumor staging and the early clinical detection of prostate cancer [64]. However, Cvjetkovic et al. reported that centrifugation parameters, such as the use of oscillating buckets or fixed-angle rotors and the duration of centrifugation, can affect the yield and purity of isolated vesicles. Their results suggest that appropriate prolongation of the centrifugation time can result in higher protein and RNA yield in exosomes, while rotational speed alone cannot predict the exosome pill-forming capacity [65].

In addition, UC can cause mechanical damage to vesicles due to the high centrifugal force, which causes a collision between vesicles and the solid-bottom vessel. This can reduce the purity of isolated exosomes and lead to the presence of some contaminant protein aggregates due to vesicle heterogeneity [66, 67].

### Density gradient ultracentrifugation

Density gradient centrifugation (DGC) is a modified ultracentrifugation method based on the size, shape, mass, and density of EVs. In a density gradient solution, when the centrifugal force of each particle is balanced with the buoyant force, different components accumulate at different positions in the top-to-bottom gradient due to their different densities [17, 68]. Accordingly, exosomes can be separated from other components in the sample. Generally, sucrose or iododiol is used to generate the density gradient [69]. Arab et al. used differential centrifugation and additional Optiprep^™^ density gradient ultracentrifugation to extract EVs released from Daphnia primordial microglia (Fig. 2b). They determined the protein content of the isolated EVs using mass spectrometry and found that the use of DGC eliminated contaminants and limited the effects of co-separating protein aggregates and other membrane particles present during the separation [70]. Iwai et al. used DGC to isolate EVs from human saliva and compared them to those isolated from a cell culture supernatant. They found that the volume and density of saliva EVs were 47.8 ± 12.3 nm and 1.11 g/mL, respectively, while those of cell EVs were 74 ± 23.5 nm and 1.06 g/mL, respectively. Thus, the volume of saliva EVs was lower and their density was higher [71].

DGC requires separation across different gradients, and time is required to reach equilibrium at each point in the gradient. Hence, this process is relatively time-consuming and time-sensitive, but the operation is relatively simple.

### Size exclusion chromatography

Size exclusion chromatography (SEC) is a separation technique based on differences in particle size. It is also known as gel filtration. The stationary phase in the column consists of porous beads (e.g., Sephadex, Sepharose, Sephacryl, and Biogel P). When the sample is added, large particles cannot enter the pores and are eluted out, being retained in the column for a shorter period of time. Meanwhile, small particles enter the pores, and their rate of elution reduces significantly. Thus, separation is achieved [72]. Marta et al. described how SEC can be used to isolate EVs from different biological fluids. A syringe or an empty column with a filter can be used to set up the SEC while placing a nylon mesh at the tip of the syringe. This can be followed by the addition of buffer to wet the filter and prevent air bubbles. Subsequently, the required volume of gel filtration matrix can be added, filled with elution buffer, wetted, and finally used (Fig. 2c) [73]. Foers et al. used SEC for EVs isolation from human synovial fluid and demonstrated that SEC can deplete the contaminants remaining after EVs concentration by ultracentrifugation. Moreover, using high-resolution mass spectrometry analysis, they found that proteinase K successfully removes fibronectin and other extracellular proteins [74]. The isolation of EVs from blood is difficult owing to the presence of lipoprotein particles. Hence, Karimi et al. combined SEC with a density cushion to separate lipoprotein particles from EVs while reducing contamination with lipoprotein particles by 100-fold, improving the purity of EVs [75]. Guan et al. compared SEC and UC in detail with regard to the extraction of exosomes from urine and found that SEC was superior to UC in recovering exosomal particles and proteins, with more purified exosomes being extracted. In contrast, during UC, rupture and incomplete precipitation occurred, resulting in lower recovery of exosomal proteins and significant loss of exosomal particles. The exosomes purified using SEC were compared with those obtained from EA.hy926 and HCV29 cell lines and showed a high internalization capacity at 4–6 h after co-incubation [76].

Therefore, compared to those extracted with UC, the EVs extracted using SEC have higher purity. However, SEC is more operationally challenging and requires additional equipment.

### Ultrafiltration

The principle of ultrafiltration (UF) is based on molecular size [77]. This process is similar to conventional filtration methods in that larger particles are retained by the filter while smaller particles pass through one or more membranes with different pore sizes or MWCOs (molecular weight cut-offs) [68, 78]. Tangential flow filtering (TFF) is also based on this principle and is an advanced form of UF. Paterna et al. used TFF to isolate EVs from microalgae, using cutoff values of 650, 200, and 20 nm (Fig. 2d), To improve sample purity and yield, they evaluated different technical parameters and conditions to improve EVs separation. The optimized TFF-based bioprocess was found to be suitable for large-scale production [79]. Busatto et al. compared UC and TFF with regard to EVs isolation from MDA-MB-231 breast cancer cell cultures. They found that TFF was superior in terms of yield and the removal of individual macromolecules and aggregates [80]. He et al. developed a method to optimize UF by introducing a 0.22µm filter and a dialysis membrane with an MWCO of 10,000 kDa to remove extracellular microbubbles larger than 200 nm [81]. Parimon et al. reported the isolation of EVs from bronchoalveolar lavage fluid using centrifugal UF with 100 kDa MWCO nanomembrane filtration unit and found that this process provided a smaller and more homogeneous distribution of EVs than UC and DGC [82]. Cardoso et al. reported that UF and SEC increased the ability to recover small EVs (sEVs) per ml of media by approximately 400 times compared to UC [83].

However, when UF is used, the membrane is prone to clogging as well as shear forces, which can destroy EVs during filtration. Nevertheless, the operation is simpler.

### Field-flow fractionation

The separation device used for field-flow fractionation is a thin, flat channel with a height of 50–500 μm. A force field is applied in a direction perpendicular to the sample flow and is subsequently focused on one side of the channel wall, where particles remain at different distances from the wall owing to their different sizes. Smaller particles farther from the sidewall get eluted before larger particles. There are different types of external fields, such as electric, gravitational, temperature, and cross-flow fields, which can be used to separate samples according to their biophysical properties [84, 85]. Of these, the most widely studied is the cross-flow field in asymmetric flow-field flow fractionation (AF4). Yang et al. reported that AF4 can be used to separate EVs from high-density lipoprotein (HDL) and lipoprotein particles (LDL) in human plasma with high purity and reproducibility. Moreover, they found that human EVs showed a higher concentration in human plasma than in equal volumes of paired serum samples, and the individual variability in the amount of EVs in human plasma was independent of age and sex. Finally, they optimized the AF4 technique and sample preparation process parameters [86]. Kang et al. used flow field-flow fractionation to isolate concentrated exosomes from Hb1.F3 immortalized human neural stem cells (HMSCs) based on their different hydrodynamic diameters (Fig. 2e) [87]. Yang et al. used the flow field flow grading method to separate urinary exosomes according to their size, and found that exosomes in prostate cancer patients were nearly twice the size of healthy individuals [88].

However, in this method, the sample volume during separation is small, which does not allow for large-scale separation and extraction. Moreover, prior fractionation and concentration are required. Nevertheless, the technique has high reproducibility and resolution.

### Precipitation method

The polymer precipitation method involves mixing the relevant biofluid with a polymer solution, followed by centrifugation at a low speed to promote exosome precipitation [60, 89] and obtain exosomes (Fig. 2f) [60]. Ludwig et al. added 50% polyethylene glycol (PEG) of different molecular weights to achieve final concentrations of 6, 8, 10, 12, and 15% PEG and 75 mM NaCl. After optimizing the method, they finally chose to add PEG 6000 and NaCl to achieve a concentration of 10% PEG and 75 mM NaCl. After incubation for 8 h, a significant amount of bovine serum proteins could be removed. However, studies show that EVs samples prepared with PEG may still retain a certain percentage of non-EV-related molecules [90]. Juan A et al. reported that the precipitation of exosomes from the cell culture medium using PEG prior to the SEC step can improve the resolution of conventional SEC methods because PEG precipitates soluble proteins. Moreover, the combination of polymer-based precipitation with SEC (Pre-SEC) methods can help in separating individual cell types secreting EVs isoforms. Today, commercial kits based on polymer co-precipitation are available for EVs isolation [91]. Jenni et al. used the miRCURY™ Exosome Isolation Kit to obtain abundant EVs-specific miRNAs from plasma. They found that the precipitation-based method was not sufficient to purify the EVs-containing miRNA cargo from plasma. Although a portion of vesicle-free miRNAs could be removed, vesicle-free miRNAs remained predominant in plasma EVs precipitates isolated by this method [92]. Romero et al. compared the performance of UC, the PEG method, and two commercial kits (Exoquick^®^ and PureExo^®^) in the isolation of gDNA-EVs from healthy donor blood. They found that the PEG method could increase gDNA yields and reduce cost and time [93].

In addition to polymer-based precipitation, charge-based precipitation can be used for EVs separation. Deregibus et al. used positively charged fish sperm proteins to induce EVs precipitation in the serum, saliva, and cell supernatants. In their study, EVs resuspension was facilitated when fish sperm proteins were precipitated using 35,000 Da PEG, and the recovery of precipitated EVs was more efficient than that of EVs obtained via ultracentrifugation using charge. The precipitation method avoids the need for expensive equipment and is suitable for the isolation of EVs from small biological samples [94]. Tan et al. proposed the use of ammonium sulfate for salting to isolate EVs from skim milk, achieving purity and yield comparable to those of UC, while EVs isolated using the ExoQuick kit were of lower purity. And they also verified the relevant function of the EVs as therapeutic carriers [95].

In the precipitation method, contamination of the polymer may reduce purity. Although the kits are expensive, they are easy to handle and readily available.

### Microfluidics

The separation and quantification of EVs via microfluidic chips have received widespread attention due to advantages such as a small sample volume, fast detection speed, and easy implementation of multi-channel detection [96]. Chen et al. designed a membrane-based microfluidic platform for EVs separation and counting using two membrane filters for rapid EVs isolation and quantification from blood. For the first time, a 0.2 μm polycarbonate membrane was used for stirring-enhanced filtration to separate small EVs, achieving a separation rate of over 99%. CD63 immunostaining on alumina membranes showed fluorescent-labeled CD63 + EVs, which could be counted under a microscope. The exosomal protein expression of individual EVs could be estimated by analyzing the fluorescent spot size distribution [97]. Gwak et al. created a microfluidic platform based on affinity capture. It consisted of two microfluidic chips: a horseshoe-shaped mouth mixer (HOMM) unit and a fish-trap-shaped microfilter unit (fish-trap) for capture and elution purification, respectively. These chips could be used in combination or operated separately, and the capture, enrichment, and release of EVs could be completed in 5 min (100 µL of sample) (Fig. 2g) [98]. Han et al. developed a microfluidic two-phase aqueous system (ATPS) for EVs separation. The ATPS device had three inlets and three outlets, forming two interfacial layers of two-phase aqueous flow, with PEG and dextran ATPS as the microfluidic channels. The device could recover up to 83.4% of EVs from the EVs–protein mixtures and remove up to 65.4% of impurities [99].

The separation of EVs via microfluidics is a relatively new method developed in recent years. It is gradually attracting attention from researchers due to the advantages of automation, low sample requirements, and the high-throughput nature of the procedure. However, issues regarding large-scale applications need to be addressed (reducing the complexity of fabrication and operation).

### Affinity-based methods

EVs isolation based on chemical affinity has been extensively studied. Affinity-based methods can be divided into two broad types: targeted EVs capture and non-targeted EVs capture. For the targeted capture of EVs, various biomolecules on the surface of EVs are targeted using a combination of high-affinity antibodies, aptamers, and peptides. Meanwhile, non-targeted capture uses lipid probes, phosphatidylserine (PS), and TiO_2_ to extract EVs based on the high affinity of lipids on the EVs surface [17]. The use of conventional spherical immunomagnetic beads can result in a lower concentration and recovery efficiency due to the smooth surface and rigid interfacial modifications of the beads. Sun et al. proposed a new technique using lipid labeling and magnetic beads by first inserting a lipid motif DSPE-PEG1000-TCO, which labels EVs in plasma, and then using bioorthogonal click chemistry to immobilize TCO-labeled EVs onto tetrazine (TZ)-grafted microspheres (clicklets). The EVs on the clicklets were then separated by centrifugation, and finally, the mRNAs in the EVs were analyzed using reverse-transcription digital PCR (RT-dPCR) (Fig. 2h). Unlike immunoaffinity-based EVs labeling, which is limited by the number of specific antigens (CD63, CD81, CD9) on the EVs surface, lipid-based EVs markers are independent of EVs surface antigens. The simultaneous combination of these markers with RT-dPCR allowed for the quantification of oncogenic changes in Ewing sarcoma and pancreatic cancer, demonstrating its potential clinical value in monitoring treatment responses and disease progression [100]. Cheng et al. developed immunomagnetic hedgehog particles (IMHPs) to capture and release exosomes. These particles were used to capture exosomes from MCF-7 cells, and the capture rate was as high as 91.7%. In contrast to exosomes obtained via UC, the exosomes obtained through this method maintained their structural integrity and showed good biological activity [101]. Yang et al. exploited the phosphatidylserine-rich surface of exosomes and immobilized peptide ligands on SiO_2_ microspheres to induce specific interactions and isolate exosomes, and isolated exosomes from serum using this method [102]. Brambilla et al. proposed the use of a DNA-directed immobilization (DDI) strategy for the isolation of EVs via the immobilization of anti-CD63 antibodies bound to vesicles on magnetic particles. That is, the surface and antibody were functionalized using complementary oligonucleotides for the release of EVs via the DNAse I-catalyzed enzymatic cleavage of the double-stranded DNA linker. Conventional methods are based on antigen–antibody destruction and alterations to their physical properties by heating or ultrasound, and treatment with organic solvents, bases, and other chemical methods can damage EVs. However, the proposed reversible DNA link could allow the release of EVs following enzymatic cleavage, overcoming this problem while enabling enhanced affinity for anti-CD63 antibodies [103]. Thuy et al. prepared a chimeric nanocomposite consisting of lactoferrin-coupled dendrimer-modified magnetic nanoparticles based on a combination of electrostatic interaction with the EVs surface, physical adsorption, and biological recognition to isolate EVs without the need for centrifugation and antibody affinity. The EVs could be separated from cell cultures and clinical specimens within 30 min, but could not be distinguished from other types of EVs [104].

### Other separation methods

Siwoo et al. separated EVs from plasma using electrophoretic migration and porous membranes, which consisted of three flow channels formed by a membrane juxtaposed between two electrodes. The sample moved horizontally through the tangential flow and migrated vertically under an applied voltage. However, negatively charged EVs sized > 30 nm could not pass through the pores and accumulated on the membrane, which was subsequently washed with PBS to collect EVs [105]. Zhang et al. separated EVs from plasma lipoproteins using agarose gel electrophoresis, a method that is based on the differences in EVs size and zeta potential. They demonstrated that the morphology of EVs recovered via electrophoresis was consistent with that of typical EVs [106]. Naohiro et al. described an efficient method for exosome preparation using anion exchange, where the cell supernatant was first separated by a 0.22 μm retention filter membrane and EVs were subsequently eluted using an anion exchange column layer [107]. This method proved to be most suitable for the preparation of GMP-compliant EVs for clinical use.

In summary, the current methods for EVs separation are diverse. However, each method has its advantages and disadvantages. Regardless of the methods, it is important to consider whether the separated EVs are complete and whether the separation and purification can be optimized by combining different techniques. Accordingly, high-purity EVs could be isolated efficiently while removing unwanted substances, ensuring safety, and minimizing costs to enable large-scale operations.

## Extracellular vesicles loading

As drug delivery carriers, EVs can be loaded with therapeutic drugs, including nucleic acids and chemotherapeutic drugs [108]. They are a promising delivery agent for carrying exogenous drugs due to their low immunogenicity and good biocompatibility. The methods of drug loading can be roughly classified as drug loading before isolation (pre-loading) and drug loading after isolation (post-loading). The characterization, loading rates, and functions of EVs loaded using different methods, such as incubation, ultrasound, and transfection, are summarized in Table 3.

**Table 3:** Loading methods for extracellular vesicles

EVs loading	Source	Loading methods	Size (nm)	Zeta potential(mV)	Cargos	Loading efficiency	Functions	Refs.
Pre-loading	ADMSCs	Incubation	sEV-CUR: 74.05 ± 2.52	N/A	Curcumin	82.26 ± 5.25%	Excellent anti-oxidative and anti-apoptotic capacity; Favorable bioavailability; Controlled release	[110]
	HEK293T	Incubation	EVs (ICG/PTX): 149.9 ± 5.2	-30.2	ICG/PTX	ICG: 60.7%; PTX: 51.9%	Simultaneous therapy and high accumulation at the tumor site; High encapsulation efficiency and cellular uptake; Photo-stability and storage stability	[111]
	HL-60; dHL60; MCF-7; THP-1	Infection	HL-60: 170.5 ± 49.4; dHL-60: 246.8 ± 19.5	N/A	Penicillin/ PTX /MCP-1/MiR-16/Cas-9-GFP/Cas9	N/A	High encapsulation efficiency and production efficiency; Low immunogenicity	[112]
	MSCs	Infection and incubation	60–150	N/A	CTX/TRA-IL	15.43 ± 0.44%	Synergistic effects and few side effects	[113]
	ADSCs	Infection	30–150	N/A	NT-3 siRNA	N/A	Stable and functional delivery	[114]
	HUVECs	USMB	N/A	N/A	CTG/BSA-FITC	N/A	High encapsulation efficiency and improved EVs production	[115]
Post-loading	RAW 264.7	Incubation	100–200	N/A	HA/CV/D-OX	N/A	Polarization to M1 macrophages; High cellular uptake; Excellent antitumor effect	[117]
	THP-1	Incubation	A15-Exo: 94.1 ± 104.4	A15-Exo: − 9.68 ± 0.29	DOX	NA	Targeting ability; high yield; efficient release	[118]
	A549	Incubation	DOX/LND-16k: 93.2 ± 24.2; DOX/LND-120k: 70 ± 11.1	DOX/LND-16k: -15.2; DOX/LND-120k: -15.9	DOX/LN-D	DOX/LND-16k: 4.16 ± 1.9%; DOX/LND-120k: 2.77 ± 0.35%	Excellent anticancer effect (DNA damage, ATP inhibition, and ROS generation)	[119]
	Macrophage	Sonication	115.0 ± 8.3	N/A	TPP1	N/A	High loading capacity; Sustained release; Bio-inspired; Non-viral and favorable stability	[243]
			DOX: 162.2 ± 1.6; PTX: 129.4 ± 2.3	N/A	DOX/PT-X	N/A	Superior intracellular accumulation and drug accumulation in cancer cells; Low immunogenicity and favorable stability	[120]
	hMSCs	Electroporation	~ 210	~ -10	GPX4 siRNA	16.6%	Magnetic targeting; BBB penetration ability; Synergistic ferroptosis therapy; Good biocompatibility and safety	[123]
	4T1	Extrusion	MSNs: 125 ± 15; E-MSNs: 150 ± 11	MSNs: 20.5 ± 1.2; ID@MS-Ns: -5.8 ± 1.5; ID@E-MSNs: -28.9 ± 3	ICG/DOX	N/A	High cellular uptake and long-term retention; Synergistic chemo-photothermal therapy; High purity; Favorable biocompatibility	[125]
	MSCs	Extrusion	135.9–194.9	-7.23	Curcumin	75.53%	Relieves neuroinflammation	[126]
	4T1	Extrusion	173	N/A	DOX	7.4%	Prominent biocompatibility; Synergistic photothermal properties; Controlled release drug; Good stability	[127]
	U87; U251	Saponin	U87-sEVs: 76.71 ± 21.7; U251-sEVs: 79.11 ± 28.77	~ -12.5	DOX	N/A	Excellent anti-oxidative and anti-apoptosis ability; Favorable bioavailability; Controlled release; Outstanding stability	[129]
	U251-GMs; SF7761- GMs	Microfluidics	U251GMs: 150 SF7761 GMs: 100	N/A	DOX	U251GMs: 31.98%; SF7761 GMs: 19.7%	Homing effect; Simple; Efficient setup; Adjustable condition	[132]
	Human plasma	Acoustofluidics	N/A	N/A	DOX	~ 30%	High loading efficiency; One-step process; Rapid encapsulation	[133]

### Pre-loading

In a nutshell, the “Pre-loading” method involves cargo loading before EVs isolation and typically has useful therapeutic effects [109]. When research on EVs first started, the drug-loading efficiency and encapsulation methods of EVs attracted extensive attention. During cell growth, cells continue to communicate with each other via EVs. During this phase, drugs can be taken up and secreted by the cell using these vesicles. As a result, the drug is loaded into the vesicles. The most common pre-loading methods are incubation, infection, and ultrasound combined with microbubbles (USMB).

**Fig. 3:**
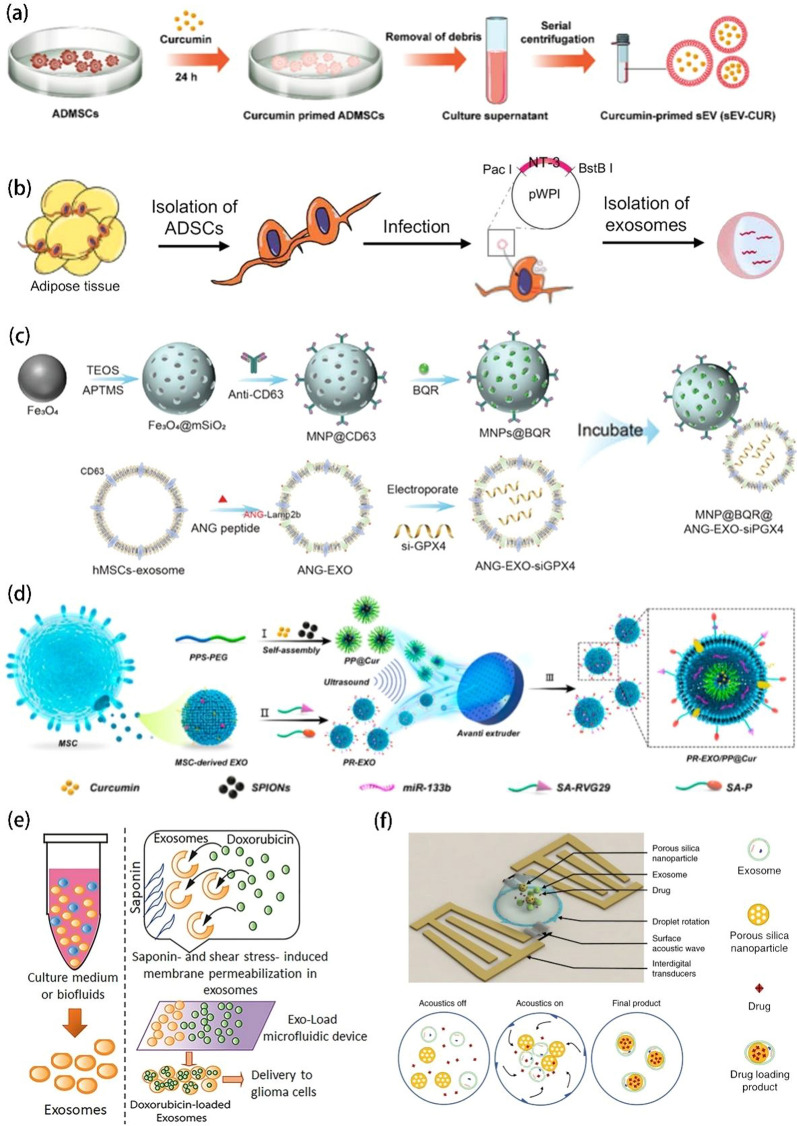
Methods for loading EVs. **a** Process of curcumin-primed ADMSC-derived sEV-CUR collection. Reprinted with permission from Ref [110]. **b** Process of NT-3 mRNA encapsulation into ADSC-derived exosomes. Reprinted with permission from Ref [114]. **c** Process of PR-EXO/PP@Cur preparation. Reprinted with permission from Ref [123]. **d** Process of the MNP@BQR@ANG-EXO-siGPX4 preparation. Reprinted with permission from Ref [126]. **e** Process of DOX loading into exosomes using the Exo-Load microfluidic device in the presence of saponin. Reprinted with permission from Ref [132]. **f** Process of acoustofluidic drug loading. Reprinted with permission from Ref [133]

Among the various strategies for drug loading via incubation, direct incubation is the simplest method. Xu et al. fabricated a new drug delivery system called sEV-CUR. In this system, curcumin (CUR) was incubated with adipose-derived mesenchymal stem cells (ADMSCs), and sEV-CUR particles were harvested using UC (Fig. 3a). The average diameter and zeta potential of the EVs remained largely consistent following incubation, and the loading efficiency was 82.26% ± 5.25%. Notably, sEV-CUR showed excellent therapeutic function (anti-oxidative stress and anti-apoptosis ability), favorable bioavailability, controlled release, and improved stability [110]. EVs are well-known to be both hydrophobic and hydrophilic owing to their phospholipid bilayer composition. Hence, Kim et al. developed a drug delivery system called ICG/PTX- EVs. To this end, human embryonic kidney 293T (HEK293T) cells were cultured to obtain EVs, which were then mixed with paclitaxel (PTX) and ICG (ICG/PTX weight ratios of 2/1). ICG was incorporated into EVs because of its hydrophilic property, while PTX was incorporated into the phospholipid bilayer of the EVs owing to its hydrophobic nature. The average size of the EVs and absolute value of the zeta potential showed a slight increase after incubation with ICG and PTX. Further, the encapsulation efficiencies of ICG and PTX were ≈ 60.7% and ≈ 51.9%, respectively [111]. As delivery carriers, EVs also exhibit favorable encapsulation efficiency, cellular internalization, accumulation at tumor sites, photostability, and storage stability.

Using the transfection method, Kim et al. transfected various drugs into cells and focused on the loading capacity of EVs from neutrophil-like differentiated human promyelocytic leukemia (dHL-60) cells. The dHL-60 cell-derived EVs had higher effector functions (larger amounts and higher efficiencies), higher efficiency of EVs production, and lower immunogenicity than HL-60-derived EVs and neutrophil-derived extracellular vesicles (NDEVs). This suggests that neutrophils and neutrophil-like promyelocytic cells could serve as a platform for EVs drug-loading [112]. Qiu et al. developed an MSC-derived exosome (MSC-EXO) vector to deliver the cabazitaxel/tumor necrosis factor-related apoptosis-inducing ligand (CTX/TRAIL) combination via transfection and achieved excellent antitumor activity. The particle size of MSCTs-EXO/CTX was approximately 60–150 nm. The entrapment efficiency and drug loading were 93.7 ± 1.53% and 15.43 ± 0.44%, respectively. MSCTs-EXO-CTX showed the synergistic effects of TRAIL and CTX [113]. Further, engineered exosomes derived from adipose-derived stem cells (ADSCs) have also been used to encapsulate NT-3 mRNA and promote peripheral nerve regeneration via infection (Fig. 3b) [114].

USMB has been shown to trigger the release of EVs from cancer cells. Yuana et al. generated drug-containing EVs using this approach. The fluorescent dye cell tracker green (CTG) or bovine serum albumin coupled with fluorescein isothiocyanate (BSA FITC) was loaded into human umbilical vein endothelial cells (HUVECs) using different ultrasound acoustic pressures. At 0.6 MPa, the BSA-FITC signal intensity in HUVECs was the highest (versus no treatment and treatment at 0.7 and 0.8 MPa). The BSA-FITC signal intensity induced by USMB was higher than that in the untreated group. HUVECs loaded with BSA-FITC also released EVs containing BSA-FITC. In brief, the results showed that USMB can be used to generate EVs containing drug cargo. However, USMB is not suitable for substances that are easily quenched and trapped in the endosomal-lysosomal degradative pathway following uptake [115].

Of the pre-loading methods, incubation is the simplest. However, it is time-consuming, and the efficiency is low. Infection is the optimal choice for adding exogenous cargos including RNA, drugs, and inorganic materials into EVs, but it is associated with operational challenges and a high cost. USMB is a good method for producing EVs containing cargo, but one of its disadvantages is easy entrapment inside organelles.

### Post-loading

Drug loading after isolation is considered as post-loading [116]. The loading methods used in pre-loading can also be used for post-loading applications.

For the post-loading incubation strategy, Carla et al. isolated EVs from M1-macrophages and incubated them with hyaluronic acid (HA) and/or the β-blocker carvedilol (CV). The EVs were called MM-EVs (modulated-M1 EVs). The MM1-EVs were further loaded (MM1-DOX) following incubation with DOX. MM1-EVs with HA and CA could promote macrophage polarization to the M1 phenotype and improve cellular uptake to enhance the antitumor effects of DOX [117]. Similarly, human monocytes (THP-1) were subjected to phorbol 12-mrustate 13-acetate (PMA) treatment and released exosomal A15 (A15-Exo) during stimulation. A15-Exo was incubated with DOX overnight to obtain A15-Exo/DOX. After that, A15-Exo/DOX was used to co-incubated with cholesterol-modified mi159 (CHO-miR159) to obtain the final delivery system called Co-A15-Exo [118]. Li et al. used EVs to load DOX and lonidamine (LND). Two types of EVs with different sizes (16k EVs and 120k EVs) were prepared with the application of different centrifugation forces following simple infusion. The average size and zeta potential of 16k-EVs and 120k-EVs were 93.2 ± 24.2 nm and − 15.2 mV and 70 ± 11.1 nm and − 15.9mV, respectively. Furthermore, the encapsulation efficiencies of DOX and LND in 16k-EVs were 0.81 ± 0.22% and 4.16 ± 1.9%, respectively, while those of 120k-EVs were 0.43 ± 0.03% and 2.77 ± 0.35%, respectively. Notably, DOX- and LND-loaded EVs exhibited excellent anticancer activity, while the smaller EVs exhibited greater inhibition of intracellular DNA synthesis, inhibition of intracellular ATP, and promotion of intracellular ROS generation [119].

Haney et al. used EVs from macrophages to target triple-negative breast cancer (TNBC) based on the sonication method. The EVs were applied as drug delivery carriers for PTX and DOX following incubation (DOX) or sonication (PTX). The obtained EVs-DOX and EVs-PTX were spherical in shape, with a uniform size distribution. Notably, EVs-DOX and EVs-PTX showed excellent intracellular accumulation, drug accumulation in cancer cells, low immunogenicity, and high stability [120].

Electroporation is another efficient way to load drugs into EVs. Following an electric pulse, pores are generated within the EVs membrane, allowing the entry of micro-molecules into EVs [121]. Zhu et al. loaded DOX into exosomes derived from lens epithelial cells (LECs) using electroporation, which effectively prevented posterior capsule clouding due to the homologous targeting of exosomes[122]. Moreover, magnetic nanoparticles (MNPs) consisting of mesoporous silica and Fe_3_O_4_ were also developed. MNPs were loaded with an inhibitor of ferroptosis compensators and modified using an anti-CD63 antibody. Thus, they could specifically bind to CD63-overexpressing exosomes derived from human MSCs. Angiopep-2 was incorporated into the exosome membrane, allowing the exosomes to cross the BBB and enter glioblastoma cells. The exosomes were loaded with a small interfering RNA against GPX4, a compensator of ferroptosis, via electroporation (Fig. 3c) [123]. Tsai et al. engineered MSC-derived sEVs to express FGL1/PD-L1 on their surface, mixed them with FK506, diluted them with PBS, and placed them in electroporation cups. They then electroporated the EVs using electroporation equipment at 300 V and 150 µF. This step was followed by incubation on ice for 30 min for membrane pore recovery, and the mixture was finally centrifuged to obtain samples [124].

To adopt the extrusion method, Tian et al. developed a 4T1 cell-derived exosome-camouflaged porous silica nanoparticles (E-MSNs), loading ICG and DOX into E-MSNs to form ID@E-MSNs. Coating with exosomes could effectively enhance the cellular uptake of ID@E-MSNs, promoting long-term retention in vivo and improving biocompatibility due to the high purity of the exosomes [125]. In other studies, a combination of ultrasound and extrusion has also been reported. Peng et al. developed a self-oriented nanocarrier (PR-EXO/PP@Cur) in order to enhance drug accumulation at the action site. This nanocarrier was developed by mixing a PR-EXO (RVG29 Peptide and Penetratin-Modified Exosome) solution with PP@Cur (PPS-PEG@Cur Micellar) via ultrasonic oscillation followed by extrusion through a mini-extruder (Fig. 3d) [126]. Exosomes derived from 4T1 cells were also used to mimic Fe_3_O_4_ magnetic nanoparticles and simultaneously load DOX. The Fe_3_O_4_@Exo NPs showed prominent biocompatibility, controlled drug release, and a synergistic photothermal-chemotherapeutic effect [127].

In some surface treatment methods, substances such as saponin, Triton, and DMSO are used to modify the surface membranes of the EVs and increase their permeability through pore formation [128]. Guo et al. developed a cargo elimination strategy to eliminate the original content from tumor-derived sEVs. Saponin treatment could effectively remove the original cargo (like proteins and RNAs) from GBM-sEVs. These treated sEVs did not promote tumor growth. Furthermore, they compared three methods (saponin, sonication, and freeze-thaw) and found that saponin treatment and sonication provided the highest protein and RNA removal efficiency, while the removal efficiency achieved through freeze-thaw cycles was far lower. In addition, they successfully achieved synchronous cargo elimination and drug loading [129]. Cao et al. mixed ICG and PTX with EVs in DMSO/PBS [4% (v/v)] and incubated the mixture for 2 h at 4 °C in the dark to achieve successful loading [130]. Cao et al. also successfully loaded ICG and folic acid (FA) into exosomes using the same method [131].

Some devices, such as microfluidics and acoustofluidics devices, can also be used for post-loading. A microfluidics device could effectively improve the loading efficiency of DOX into U251-GM-derived-exosomes from 7.86 to 31.98% by adjusting the flow rate and microchannel types. The DOX-loaded GMB-derived exosomes prepared accordingly showed a homing effect (Fig. 3e). In the process of DOX loading, saponin was used as a permeabilizing agent to change the properties of the cellular membrane, exerting a combined effect in concert with the shear stress-induced stimulation in the microchannel [132]. Acoustofluidics devices with a combination of porous silica nanoparticles, exosomes, drugs, droplet rotation, surface acoustic waves, and interdigital transducers can simplify drug loading and exosome encapsulation into a one-step process, improving the efficiency of drug loading (Fig. 3f). The final product consists of the drug, porous silica nanoparticles, and an inside-out exosome membrane, and the device-derived exosome encapsulation can significantly enhance endocytosis in vitro during cell-intake experiments. Furthermore, the device can encapsulate drug-containing nanoparticles of different morphologies into exosomes [133].

In the post-loading strategy, the incubation and sonication methods are similar to those used in pre-loading strategies. Electroporation is a rapid-loading method that acts by creating physical pores in the membrane. However, it can damage the integrity and flexibility of the membrane. Extrusion is a convenient choice, but it provides insufficient encapsulation. Device-based methods required an advanced facility and are not universally feasible.

In conclusion, the various cargo loading methods can be divided into pre-loading and post-loading strategies. EVs loading has multiple advantages, including synergetic effects, high loading and encapsulation efficiency, favorable stability, and functional delivery. However, different methods affect EVs from the same source in different manners. In general, the pre-loading method (incubation) is easier but more time-consuming. External interventions such as ultrasound, electroporation, and extrusion improve loading efficiency while causing a deterioration in stability. The saponin-mediated cargo elimination strategy is a good reminder that the original contents of EVs should be eliminated before loading new substances. Of note, the two advanced devices mentioned above provide a useful tool for the fusion of EVs with other agents (e.g., DOX), due to their prominent properties, including simple operation, adjustable conditions, rapid encapsulation, and high loading efficiency.

## Surface modification of extracellular vesicles

EVs are biocompatible and have an ideal natural structure and hydrophilic core. Hence, they are increasingly being used as drug carriers or therapeutic agents and are expected to serve as valuable nanocarriers for clinical use. Although their surface expresses intact transmembrane proteins (CD81 and CD91) and integrins (CD51 and CD61) with homing and targeting functions, their targeting ability remains weak. EVs show heavy accumulation in the liver and spleen and are subsequently cleared by macrophages. Therefore, their modification can facilitate the delivery of cargo to target cells [134–137]. The current surface functionalization strategies can be divided into two categories: endogenous and exogenous. The methods used for modifying EVs, related strategies, sources of EVs, and functions after modification are summarized in Table 4.

**Table 4:** Methods and strategies for the modifications of EV surfaces

Surface modification	Strategy	EVs source	Functions	Refs.
Genetic engineering	Infection with PGMLV-PA6 virus expressing both the CXCR4 protein and GFP	MSCs	Allowed more MSC-derived exosomes to nest around the target region	[139]
	Transfection via the pCDH-GFP vector	HuCMSCs	Decreased ATP concentration; increased adenosine levels; and reduced spinal cord inflammation	[140]
	Transfection via the recombinant adenoviral vector GFP-CTF1 encoding CTF1	BMSCs	Increased proangiogenic activity and the rates of successful pregnancy outcomes	[141]
	Transfection via a pCDNA-MEG3 vector	OS cells	Inhibition of osteosarcoma growth	[143]
	Introduction of pcDNA3.1(-)-RGD-Lamp2b into cells by electroporation	HEK293T	Enhanced tumor site targeting	[145]
	Transfection via a miR-31-5p lentiviral vector	HEK293	Promoted the healing of diabetic wounds	[144]
	Co-transfection of a reporter plasmid and miR-181b mimics using Lipofectamine	Human umbilical cord mesenchymal stem cells (HuCMSCs)	Enhanced M2 polarization; inhibited inflammation; and promoted osseointegration	[244]
	Transfection via an XStamp-PDGFA lentiviral vector	Neural stem cells (NSCs)	Improved potential for CNS injury targeting	[245]
	Transfection via an LV-iRGD-Lamp2b lentiviral vector	Human cord blood MSCs (cbMSCs)	Enhanced targeting to tumor sites	[246]
	Transfection via a Lenti-XStamp-PDGFA lentiviral vector	Neural stem cells (NSCs)	Enhanced targeting efficiency for central nervous system lesions	[247]
	Transfection via the pRBP-Lamp2b-HA-hygro vector using Lipofectamine 2000	HEK293 cells	Anti-inflammatory effects	[248]
	Transfection via the iRGDC1-EGFP-Lamp2b virus	HEK293T	Enhanced tumor targeting	[249]
Bioorthogonal chemistry	The azide groups were bioorthogonally labeled with DBCO-Cy5 via bioorthogonal click chemistry	A549	Exosome tracking and imaging	[148]
	DBCO reacted with azide or azide-containing methionine analogs via bioorthogonal click chemistry	B16F10	Regulation of exosome composition and binding of exosomes for intracellular delivery	[147]
	DBCO-Exo was linked to a c(RDGyK) peptide with an azide moiety via copper-free click chemistry	MSCs	Improved targeting of lesion sites	[149]
	Copper-free click chemistry was used with AlexaFlour®488 (AF488)-azide	PANC-1 B16F10 HEK293	Achieved quantification of intracellular tracking and intracellular uptake	[150]
Physical modification	Membrane extrusion method	HCC	Enhanced targeting ability and improved siRNA transfection efficiency	[153]
	Lipid membrane fusion	Sf9 insect cells	Enhanced targeting capabilities	[154]
	Membrane extrusion method	SKOV3-CDDP	Enhanced targeting capabilities	[155]
	Membrane extrusion method	L-929	Depleted cells and homing effects	[156]
	Membrane fusion technology using the freeze–thaw method	CT26	Allowed immune evasion, enhanced targeting ability, and acted as a drug carrier	[157]
	PEG-mediated membrane fusion	HUVECs	Widely used in studies on the mechanism of membrane fusion	[158]
	Membrane extrusion method	J774A.1	Enhanced targeting ability and acted as a drug carrier	[159]
	Incubation-mediated membrane fusion	HEK293FT	Enabled efficient wrapping of clustered regularly interspaced short palindromic repeats (CRISPR)/CRISPR-associated protein 9 (Cas9) in exosomes	[250]
	Incubation	HEK293T	Improved targeting capabilities	[251]
	Electrostatic interaction	MSCs	Targeted hepatocyte asialoglycoprotein receptors	[160]
	Extrusion method	Bone marrow MSCs	Improved targeting ability and promoted angiogenesis	[161]
Chemical modification	Bioconjugate chemistry	Human leukemia monocytic cell line (THP-1)	Promoted blood–brain barrier penetration and improved targeting	[164]
	Copper-free click chemistry	M2-BV2	Provided rapid and effective recruitment and differentiation transformation of neural stem cells	[165]
	Cycloaddition reaction of sulfonyl azide	U-87 MG	Improved targeting	[166]
	Hydrophobic insertion method	ADMSCs	Improved targeting	[167]
	Lipid insertion method	BMSCs	Improved targeting	[168]
	PDA self-polymerization and thiol-Michael addition reactions	L929	Facilitated fluorescent labeling	[169]
	Hydrophobic interaction (lipid insertion method)	MSCs derived from human induced pluripotent stem cells (iPSCs)	Improved targeting	[170]
	Thiol-maleimide click reaction	Milk	Facilitated fluorescent labeling and improved targeting	[171]
	Phospholipid insertion method	Primary human adipose-derived stem cells	Improved targeting	[252]
	Phospholipid insertion method	Milk	Improved targeting	[253]
	Phospholipid insertion method	HepG2	Photothermal effects and improved targeting	[254]
	EDC/NHS chemistry	MSCs	Improved targeting	[255]
	Membrane anchoring	B16F10	Improved targeting and imaging capabilities	[256]
	Lipid-anchoring method	BMDCs	Improved specificity for T cells	[257]

### Endogenous modification

Endogenous approaches include engineering at the cellular level to produce the desired proteins and peptides via the transfection of donor cells. In addition to genetic manipulation, bioorthogonal chemistry also serves as an important tool in this strategy [138].

#### Genetic engineering

**Fig. 4:**
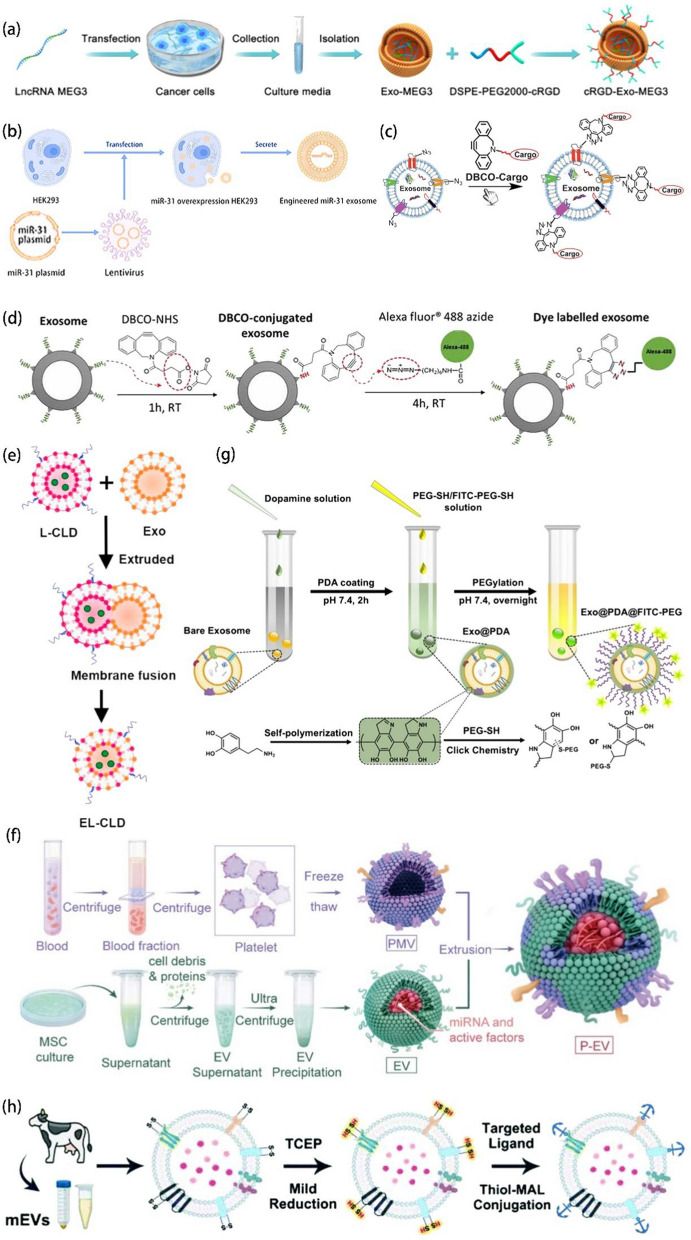
**a** Schematic diagram showing the collection and isolation of exosomes rich in lncRNA MEG3 (Exo-MEG3). Reprinted with permission from Ref [143]. **b** Schematic diagram illustrating the development of engineered miR-31 exosomes. Reprinted with permission from Ref [144]. **c** Bioorthogonal click conjugation for exosome functionalization. Reprinted with permission from Ref [147]. **d** Schematic diagram depicting the fluorescence labeling of the exosome surface using copper-free click chemistry and AlexaFlour^®^488 (AF488)-azide. Reprinted with permission from Ref [150]. **e** Schematic illustration of the procedure used to produce the EL-CLD hybrid, including the hybridization of exosomes with L-CLD using membrane extrusion. Reprinted with permission from Ref [156]. **f** Schematic diagram of P-EV preparation. Reprinted with permission from Ref [162]. **g** Schematic illustration of the PDA coating of exosomes and subsequent surface functionalization with PEG and fluorophores. Reprinted with permission from Ref [169]. **h** Schematic diagram of mEV isolation and surface functionalization via TCEP reduction. Reprinted with permission from Ref [171]

Genetic engineering involves the transfer of a foreign gene into a recipient cell after in vitro recombination, such that the gene is ectopically expressed in the recipient cell [136]. Xu et al. obtained exosomes expressing high levels of chemokine receptor 4 (CXCR4) via the lentiviral infection of MSCs. These exosomes showed more efficient aggregation at tumor sites, attacking tumor cells and showing great potential in specific targeted therapy [139]. Zhai et al. established the first CD73-engineered EVs. They transduced the target gene into human umbilical cord mesenchymal stem cells (HuCMSCs) via a lentiviral vector and isolated EVs. These EVs showed upregulated CD73 expression and reduced the inflammatory response to a large extent [140]. Zhu et al. constructed an adenoviral vector that promotes the stable overexpression of cardiotrophin-1 (CTF1) and transduced CTF1 into bone MSCs (BMCSc) using this vector at an MOI of 500. They found that the transduction efficiency peaked after 48 h [141]. lncRNA MEG3 is aberrantly expressed in many tumors, and it is knocked out in some types of cancer [142]. Huang et al. used pCDNA-MEG3 to transfect osteosarcoma (OS) cells and induce lncRNA MEG3 overexpression in their isolated exosomes (Fig. 4a). They also demonstrated that lncRNA MEG3 plays an important role in osteosarcoma (OS). No significant toxicity was observed in vivo, indicating that the therapeutic delivery system was effective and safe [143]. Using patient samples and in vitro studies, Huang et al. found that miR-31-5p was a key miRNA in diabetic trauma physiology. Hence, HEK293 cells were transfected with a miR-31-5p lentiviral vector, and engineered miR-31 exosomes were isolated to serve as a precision therapeutic agent (Fig. 4b) [144]. Zhao et al. inserted Lamp2b primers into the pcDNA3.1(-) vector between Nhe1 and BamH1 and subsequently transfected this modified vector into HEK293T cells using an electroshock method. They obtained RGD-Lamp2b-engineered exosomes, which showed greater aggregation in tumor tissues and better tumor targeting than unmodified exosomes [145].

Modifications via genetic engineering, which is directly designed for parental cells, allow the upregulation of nucleic acid expression for therapeutic purposes. However, it is costly, and insufficient transfection efficiency remains a concern.

#### Bioorthogonal chemistry

Bioorthogonal chemistry refers to the chemical reactions that can be carried out within biological systems without interfering with natural biological processes [146]. Copper-free click chemistry methods are widely used for the modification of EVs. Wang et al. were the first to propose a method to modify and functionalize exosomes using exosomal secretory cells co-cultured with azide to allow azide to bind with the chemically active sites of exosomes via a bioorthogonal reaction (Fig. 4c) [147]. Song et al. used bioorthogonal click chemistry to fluorescently label exosomes in order to achieve real-time tracking in vivo and in vitro, after first linking azide groups to the cell surface via glycometabolic engineering and then linking the fluorescent DBCO to the azide groups through bioorthogonal click chemistry. Compared with the use of DiD fluorescence-labeled exosomes, the use of in situ bioorthogonal click chemistry is more efficient and less toxic, and it has less impact on the intrinsic properties of the exosomes [148]. Tian et al. introduced DBCO into amine-containing molecules on exosomes, which was followed by copper-free click chemical attachment to c(RDGyK) polypeptides with azide moieties, improving exosome targeting via surface modification [149]. Xu et al. quantified intracellular tracking and uptake by successfully labeling exosomes with DBCO-NHS, AF488-azide, and fluorescent tags via a copper-free chemical click (Fig. 4d) [150].

Although the efficiency of copper-free click chemistry in modifying the surface of EVs is lower than that of copper click chemistry, this technique can prevent the oxidation of membrane proteins by copper ions and also improve the safety of exosomes [151].

### Exogenous modification

Exogenous modifications refer to the direct modifications of the EVs membrane and can be performed using physical or chemical methods.

#### Physical modification

Physical methods involve the use of physical forces and processes such as ultrasound, incubation, extrusion, and freeze-thaw methods to temporarily disrupt the lipid structure of the vesicles. With these methods, the vesicles self-assemble into their original structure when the forces disappear [152]. Further, the vesicles can also be modified via electrostatic interactions or weak forces between EVs and functional molecules (e.g., fusion of lipid membranes) [134]. Zhou et al. isolated tumor-derived EVs (TDEVs) from hepatocellular carcinoma (HCC) cells and subsequently hybridized them with lipid nanovesicles to obtain innovative nanovesicles (LEVs), which were generated by the fusion of TDEV membranes with phospholipids. LEVs showed better targeting abilities due to the “homing” effect and a 1.7-fold higher siRNA transfection rate than liposomes [153]. Raga et al. exploited the presence of Gp64 in the viral particles of Sf9 insect cells and their membrane fusion under acidic conditions. Sf9 insect cell EVs, which also express the functional membrane protein PD-1 on their surface and can be actively targeted by Cx43, were isolated. The fusion of Gp64-expressing PD-1 EVs with small molecule liposomes contributed to the generation of heterozygous EVs with further biomedical applications [154]. Li et al. designed a novel pH-sensitive bionanoplasmic nanosystem comprising SKOV3-CDDP-derived exosomes hybridized with cRGD peptide-modified liposomes via the membrane fusion technique. Exosomes were vortexed with liposomes after vortex sonication in a vacuum vortex and finally extruded using a 200 nm polycarbonate membrane filter. A dual-targeting effect including homologous targeting and cRGD could be achieved [155]. Sun et al. proposed the preparation of EL-CLD hybrids by the extrusion of fibroblast-derived exosomes (E) with liposomes (L) loaded with clodronate (CLD) (Fig. 4e), and the EL-CLD obtained had a good drug release potential [156]. Cheng et al. used the freeze-thaw method to mix temperature-sensitive liposomes with genetically engineered exosomes. The mixture was frozen at −80 °C for 15 min and rewarmed at 37 °C for 15 min. After three cycles, lipid membrane-fused nanovesicles (hGLV) were obtained [157]. Max et al. developed a method for fusing EVs with functionalized liposomes in the presence of PEG and retaining the natural properties of EVs. The drug delivery efficiency of the heterogeneous EVs was 3–4-fold greater than that observed with free drug and liposome delivery [158]. Sagar et al. crossed mouse macrophage-derived J774A.1 sEVs with liposomes to obtain engineered hybrid exosomes (HEs) that retain the advantages of endogenous sEVs, which target tumor sites, and liposomes, which exhibit significant flexibility for surface modification as potential drug delivery carriers. The disadvantages of both types of particles could be overcome, and their advantages could be combined, to generate an effective hybrid drug delivery tool [159]. Tamura et al. used cationic pullulan polysaccharides to modify exosomes via electrostatic interactions [160]. Hu et al. hybridized platelet membranes with stem cell-derived exosomes to enhance binding and accumulation in damaged tissues [161]. Li et al. prepared platelet-like membranes via the fusion of platelet membranes with bone marrow MSC-derived EVs using extrusion for improving the ability to target the lesion site (Fig. 4f) [162].

Modification of the EVs surface by physical means avoids the introduction of foreign impurities and improves the purity of EVs while causing less damage to them. However, some complex auxiliary equipment is often required during the preparation process. Moreover, the extrusion method may lead to variations in the dimensions of EVs.

#### Chemical modification

Some functional groups are present in EVs and EVs-secreted cellular transmembrane proteins. Hence, chemical modification can be performed via covalent coupling using chemical reagents that add functional groups to the EVs surface[134]. Due to the hydrophobic nature of EVs membranes, functionalized phospholipids can also be inserted [163]. Biocoupling methods and other similar strategies also exist. Liang et al. constructed an exosome nanocarrier using biocoupling chemistry. First, DSPE-PEG2000-Mal was incubated with cells for 48 h. Subsequently, exosomes with DSPE-PEG2000-Mal were extracted, and siRNA was loaded into the exosomes using ultrasound treatment. Finally, Angiopep-2 (An2) was added via DSPE-PEG2000-Mal bridge binding, and An2-functionalized exosomes were safe and effective for the treatment of glioblastoma [164]. Ruan et al. used click chemistry to modify the surface of M2 microglia-secreted EVs with vascular targeting peptide (DA7R) and stem cell recruitment factor (SDF-1). First, the azide group was introduced onto the DA7R peptide and SDF-1 via an amide reaction. This was followed by the reaction of dibenzocyclooctyne (DBCO)-DBCO-terminated PEGylated N-hydroxysuccinimidyl ester (DBCO-PEG4-NHS) with the amino group of the EVs membrane protein to achieve EVs modification. Finally, DA7R- and SDF-1-modified functionalized EVs were prepared using a copper-free azide-alkyne cycloaddition reaction. The enhanced recruitment of neural stem cells provided new insights into the use of click chemistry EVs for disease treatment [165]. Fan et al. modified exosomes with RGE via the cycloaddition of sulfonyl azide [166]. Wu et al. obtained CREKA-functionalized sEVs (CREKA-sEVs) by inserting DMPE-PEG-CREKA into the sEVs membrane from adipose MSCs using the hydrophobic insertion method, maintaining sEVs activity while inducing therapeutic effects [167]. Rehman et al. coupled hydroxyl-capped HSPP with DSPE-PEG-MAL, and the DSPE end of the synthesized HSSP-PEG-DSPE was inserted into the phospholipid membrane of exosomes [168]. Wang et al. successfully encapsulated PDA into exosomes via the self-polymerization of dopamine (PDA), which also has catechol and amine groups and undergoes the thiol-Michael addition reaction with PEG-SH, to obtain fluorescently labeled exosomes (Fig. 4g) [169]. The anchoring of diacyl lipid tail-modified bone-targeting polypeptides to exosomal membranes by hydrophobic interaction was reported by Cui et al. [170]. Chen et al. used a mild reducing agent, TCEP, to reduce the disulfide linkage on the EVs surface and then used a thiol-maleimide coupling reaction to attach the desired fluorescent label or ligand to the EVs surface (Fig. 4h) [171].

Functionalization using chemical methods is relatively convenient and fast. However, whether the introduction of new chemicals will destroy the integrity of EVs and whether the chemicals themselves have good biocompatibility warrants further investigation.

In conclusion, the functionalization of EVs aims to effectively enhance their desired effects and compensate for the deficiencies of natural EVs, improving targeting ability and drug delivery and enabling wider applications in diagnosis and therapy. However, whether the structure of EVs remains intact following functionalization remains a concern.

## Extracellular vesicles-based Nanoplatforms for therapeutics and drug delivery

The application of EVs as drugs or delivery vehicles for the treatment of cancer, neurodegenerative diseases, and regeneration is prompted by their unique biocompatibility, low immunogenicity, and stability [46]. EVs have fewer limitations with respect to the safety and feasibility of cell transplantation than cell-based therapies in the context of regenerative medicine [172]. The application of EVs as therapeutic agents or carriers in different areas of medicine is summarized in Table 5.

**Table 5:** Strategies and doses of EVs for different diseases

Treatment strategies	Diseases	Drugs	Dose of EVs in vivo	Administration	Refs.
Immunotherapy	Breast cancer	N/A	200 µg per mouse	Intravenous injection	[176]
Immunotherapy	Gastric cancer	N/A	5 mg/kg (100 µL)	Intravenous or intratumoral injection	[177]
Immunotherapy	Liver cancer	siRNA	N/A	Intratumoral injection	[178]
Immunotherapy	Pancreatic cancer	Galectin-9; siRNA	~ 10^8^ exosomes	Intravenous injection	[179]
Chemotherapy	Glioma	TMZ; DHT	N/A	Intravenous injection	[181]
Chemotherapy	Triple-negative breast cancer	DOX	N/A	Intravenous injection	[258]
Chemotherapy	Glioma	DOX	0.15 mg/mL (400 µL)	Intravenous injection	[182]
Phototherapy	Liver cancer; Breast cancer	N/A	50 mg/mL (100 µL)	Intravenous injection	[184]
Phototherapy	Gastric cancer	Proton pump inhibitor (PPI)	N/A	Intravenous injection	[186]
Gene Therapy	Triple-negative breast cancer	Anti-miRNA-21; anti-miRNA-10b	3 ~ 4 × 10^11^ NPs (150 µL )	Intravenous injection	[188]
Combination therapy	Glioblastoma	AQ4N	200 µg (100 µL)	Intravenous injection	[191]
Other	Lung cancer	Dinaciclib	200 µg	Intravenous injection	[173]
Other	Nasopharyngeal carcinoma	N/A	NPC tumor-bearing mice: 30 µg/mouse C666-1/NPC43 tumor-bearing mice: 20 µg/mouse	Intravenous or intraperitoneal injection	[194]
Other	Glioblastoma	Verrucarin A (Ver-A)	N/A	Intravenous injection	[195]
Gene therapy	Parkinson’s Disease (PD)	Glial-cell-line-derived neurotrophic factor (GDNF)	3 × 10^9^ particles/10 µL/mouse	Intranasal injection	[200]
Gene therapy	PD	Anti-TNF-α antisense oligonucleotide (ASO)	2.7 × 10^9^ particles	Intravenous injection	[202]
Gene therapy	PD	shRNA Minicircles	150 µg (100 µL)	Intravenous injection	[204]
Other	PD	N/A	N/A	Intravenous injection	[201]
Gene therapy	Bone regeneration	Bone morphogenetic protein-2 (*BMP2*) gene	50 µg (1.8 µg/µL)	In situ injection into the bone defect	[212]
Other	Periodontal disease	Minocycline	230 µg sEV	Injection into the site of defect	[209]
Other	Hair loss	N/A	200 µg Milk-exo in 100 µL saline	Intradermal injection	[210]
Other	Wound healing	N/A	100 µg (100 µL)	Subcutaneous injection	[211]
Other	Bone regeneration	N/A	50 µg/per cranial defect	Implantation into the defect	[213]
Other	Shoulder stiffness (SS)	N/A	50 µL EV (20 µg/mL or 50 µg/ml, equal to final particle from 1.2 × 10^8^ to 3 × 10^8^)	Intra-articular injection	[216]
Other	Inflammation	Piceatannol	N/A	Intravenous injection	[217]
Other	Rheumatoid arthritis (RA)	Dexamethasone sodium phosphate	N/A	Intravenous injection	[218]

### Cancer therapy

Cancer is associated with high morbidity and mortality, and traditional treatments such as surgery and radiotherapy have certain limitations (e.g., easy recurrence and severe side effects). However, some of these problems can be addressed using nano-drug delivery systems. EVs are widely used in cancer treatment because of their ability to penetrate tissues, tumor tropism, and low immunogenicity [173]. Further, as with cell membrane coating technologies, the CD47 expressed on the membrane can aid in immune evasion, preventing the engulfment of nanoparticles [174].

#### Immunotherapy

Immunotherapy has emerged as a powerful clinical strategy for the treatment of cancer. However, the key challenge in the widespread implementation of immunotherapies against cancer remains the controlled regulation of the immune system, as these therapies have serious side effects, including autoimmunity and non-specific inflammation. Thus advanced biomaterials and drug delivery systems [175], such as the use of macrophage exosomes could effectively harness immunotherapy and increase its efficacy while reducing toxic side effects. Macrophages are usually found in two phenotypes: classically activated M1 cells and alternatively activated M2 cells. The M1 phenotype of macrophages exerts antitumor effects, while the M2 phenotype exerts pro-tumor effects. Nie et al. coupled anti-CD47 with anti-Sirpα antibodies (aCD47 and aSirpα) on M1 macrophage-derived exosomes via pH-sensitive linkers. aCD47 recognizes tumor cells by identifying the CD47 on their surface and thereby actively targets the tumor. As a result, the “don’t eat me” signal disappears, leading to the enhanced phagocytosis of macrophages. Meanwhile, M1 exosomes can also transform pro-tumor M2 macrophages into antitumor M1 macrophages, while the M1-Exo synergistic antibodies exert antitumor effects [176].

**Fig. 5:**
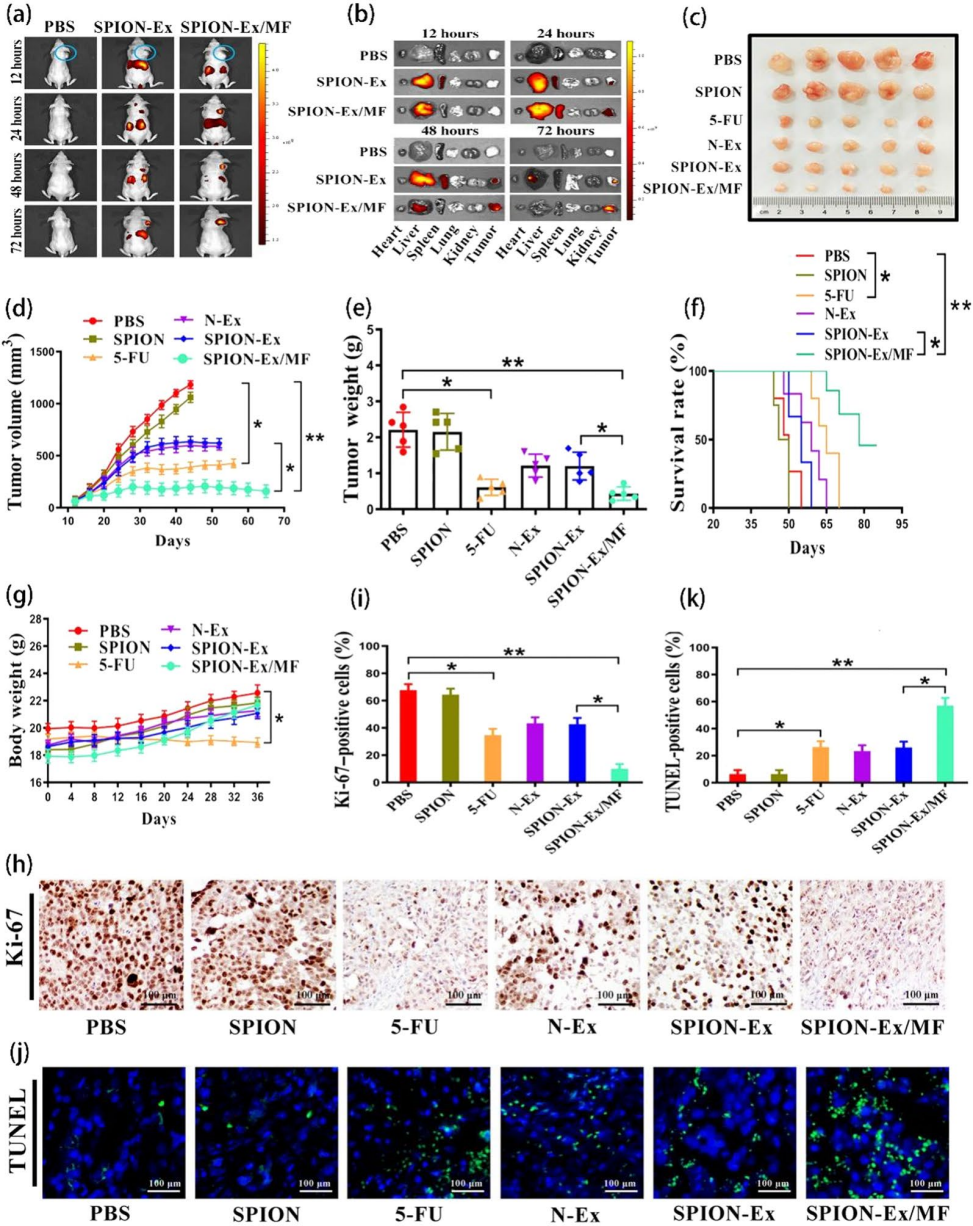
Tumor-targeting ability and therapeutic effect of SPION-Ex/MF in vivo. **a** Representative images showing the distribution of DiR-labeled SPION-Ex (5 mg/kg body weight) in BALB/c nude mice under an external magnetic field at 12, 24, 48, and 72 h after intravenous injection (n = 4 per group). The circles indicate the tumor sites. **b** Representative images showing the ex vivo fluorescence signals of DiR-labeled SPION-Ex (5 mg/kg body weight) in major organs (liver, spleen, lung, heart, and kidney) and tumors at 12, 24, 48, and 72 h after intravenous injection. **c** Representative images of subcutaneous xenograft tumors established using HGC27 cells in BALB/c nude mice. **d** Tumor growth was routinely examined, and the tumor growth curves in each group are shown. **e** Weights of harvested xenograft tumors. **f** Survival rates of mice receiving different treatments. **g** Body weights of mice at the end of the study. **h–i** Ki-67 staining of tumors from mice receiving different treatments, including PBS, SPION (2 mg/kg body weight), 5-FU (5 mg/kg body weight), N-Ex (5 mg/kg body weight), and SPION-Ex (5 mg/kg body weight), with or without MF. Scale bars, 100 μm. **j–k** TUNEL staining of tumors from mice receiving different treatments, including PBS, SPION, 5-FU, N-Ex, and SPION-Ex, with or without MF. Reprinted with permission from Ref [177]

Neutrophils are innate immune cells that directly act as cytotoxic agents against tumor cells. Zhang et al. used superparamagnetic oxidized nanoparticles (SPION) to modify neutrophil-derived exosomes (SPION-Ex) for targeted cancer therapy. They used HGC27 cells to establish a subcutaneous tumor model in BALB/c nude mice and treated these mice with DiR-labeled SPION-Ex. The tumors were imaged at 12, 24, 48, and 72 h post-treatment. SPION-Ex was found to show the strongest fluorescence at the tumor site after 72 h under treatment with an applied magnetic field (MF) (Fig. 5a). In vitro imaging findings were consistent with in vivo imaging results, and the concentration observed was time-dependent (Fig. 5b). After seven doses, SPION-Ex significantly inhibited tumor growth in vivo under an applied MF (Fig. 5c–e). Moreover, 40% of the mice survived until the end of the experimental period (Fig. 5f). The body weight of mice in the SPION-Ex-related treatment groups was significantly increased (Fig. 5g). The tumor tissues from the SPION-Ex/MF group had fewer Ki-67-positive cells and more TUNEL-positive cells (Fig. 5h–k), indicating greater rates of apoptosis. These findings demonstrated that SPION-Ex/MF treatment has a significant targeted treatment effect in vivo and shows a good safety profile [177].

Zhang et al. designed light-activatable silencing NK-derived exosomes (LASNEO) by modifying NK cell-derived exosomes with an siRNA and the photosensitizer Ce6. The NK-derived exosomes (NEO) themselves could kill tumor cells and restore the immunosurveillance function of T cells in the TME. Meanwhile, the ROS generated after light exposure could promote M1 macrophage polarization, DC cell maturation, and intracellular siRNA release, effectively realizing immunotherapy [178]. Zhou et al. used BM-MSC exosomes loaded with Galectin-9 siRNA. The exosomes were surface-modified with oxaliplatin (OXA) prodrugs to improve tumor targeting and inhibit macrophage polarization, cytotoxic T cell (CTL) recruitment, and Treg downregulation and thereby exerted antitumor immune effects [179]. Carlos et al. used tumor-derived EVs (TEVs) to create a novel tumor vaccine model by activating DC cells and thus achieved an antitumor immune response. The use of EVs from autologous tumors appeared to inhibit tumor recurrence [180].

Some EVs secreted by immune cells and cancer cells may have immunomodulatory functions. By applying these characteristics, these EVs can be used as drug delivery vehicles or to directly stimulate antitumor immune responses. However, EVs derived from tumors can both promote and suppress tumors. Thus, a better understanding of the mechanisms through which they modulate tumor activity is required to make them more effective as immunotherapy agents.

#### Chemotherapy

**Fig. 6:**
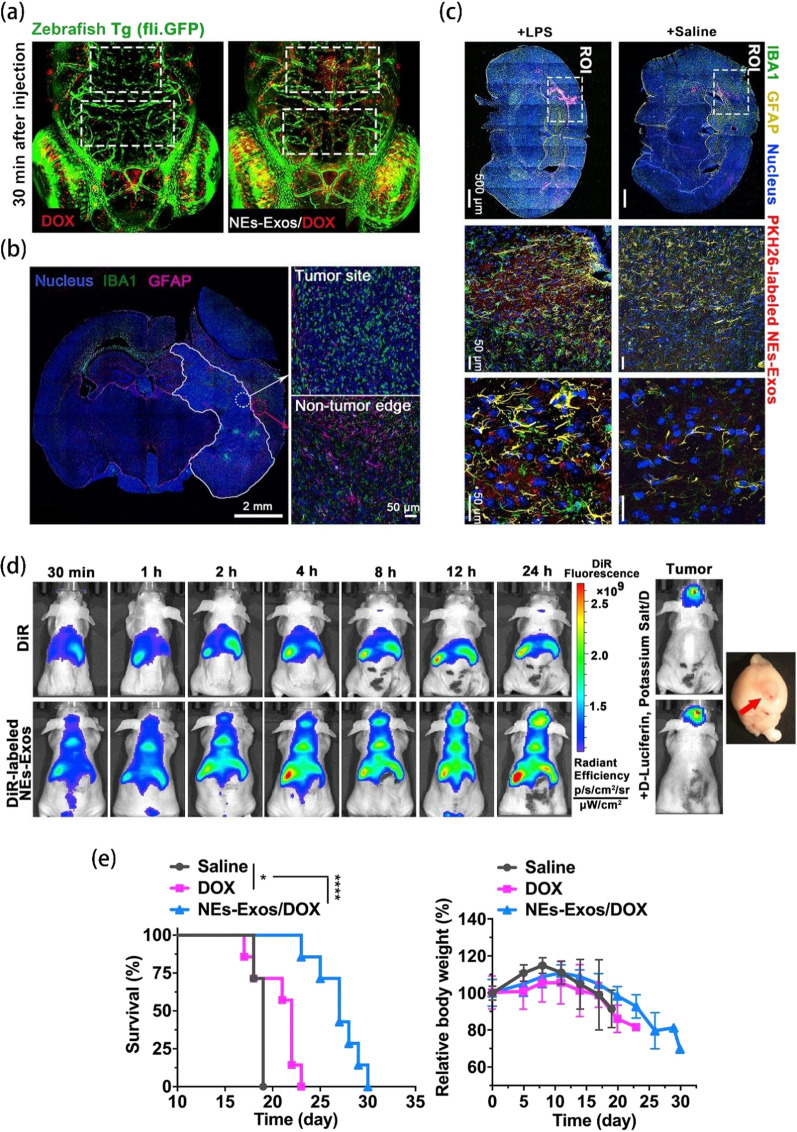
**a** Confocal imaging of DOX and NEs-Exos/DOX crossing the zebrafish BBB at 30 min. DOX and NEs-Exos/DOX were intracardially injected into the circulation of Tg (fli:GFP) zebrafish. **b** Immunofluorescence staining of IBA1 (green) and GFAP (magenta) in brain tissue. **c** In vivo distribution of PKH26-labeled NEs-Exos (red) in brain with LPS-induced brain inflammation. Brain treated with saline was considered as the control. Immunofluorescence of IBA1 (marker of microglia activation, green) and GFAP (marker of astrocytes, yellow) indicate the inflammatory condition after LPS and saline treatment. **d** Real-time fluorescence tracking of DiR and DiR-labeled NEs-Exos in C6-Luc glioma-bearing mice. **e** Body weight changes and Kaplan-Meier survival analysis of glioma-bearing nude mice. *P < 0.05, **P < 0.01 and ****P < 0.0001 indicate significant differences. Reprinted with permission from Ref [182]

Brain tumors are treated with surgery, radiotherapy, and chemotherapy. However, high recurrence rates and drug resistance limit the efficacy of these treatments. Wang et al. loaded the chemotherapeutic agent temozolomide (TMZ) and the Chinese herbal medicine dihydrotanshinone (DHT) into tumor-derived exosomes (R-EXO) because DHT can overcome the drug resistance caused by TMZ. Meanwhile, R-EXO could homologously target the tumor site, inducing the synergistic effects of chemotherapy and immunotherapy for glioma treatment [181]. Wang et al. extracted the exosomes from neutrophils (NEs-Exos) and delivered DOX for the treatment of glioma. In a Tg(fli:GFP) transgenic zebrafish model, NEs-Exos were verified to cross the BBB, as evidenced by strong red fluorescence of DOX in the brain (Fig. 6a). Subsequently, a lipopolysaccharide (LPS)-induced mouse encephalitis model was established, and significant allograft inflammatory factor 1 (IBA1) expression was detected in and around the glioma tissue. The expression of GFAP was higher around gliomas, and in contrast to the saline group, LPS-treated mice showed higher levels of IBA1 (Fig. 6b). GFAP expression was elevated in the LPS-treated mice when compared with the saline group, while the stronger inflammatory response increased the recruitment of NEs-Exos (Fig. 6c). Subsequent imaging of C6-Luc glioma-bearing mice injected with DIR showed stronger fluorescence in the NEs-Exos group within 24 h (Fig. 6d). Notably, NEs-Exos/DOX significantly prolonged the survival duration of glioma mice. The decline rate of body weight was also lower in the NEs-Exos/DOX group relative to the DOX and that in saline groups (Fig. 6e). Therefore, the use of neutrophil exosomes for DOX delivery appears to be a promising chemotherapeutic approach for the treatment of gliomas [182].

Chemotherapy is a common antitumor approach in clinical practice. However, due to its low targeting ability, it causes problems such as systemic toxicity, side effects, and a short circulation time. However, EVs can target the lesion site in their original state or after modification, effectively delivering chemotherapeutic drugs to tumor tissues and reducing adverse reactions, with low immunogenicity. Thus, they appear to be optimal carriers for the delivery of chemotherapeutic drugs.

#### Phototherapy

**Fig. 7:**
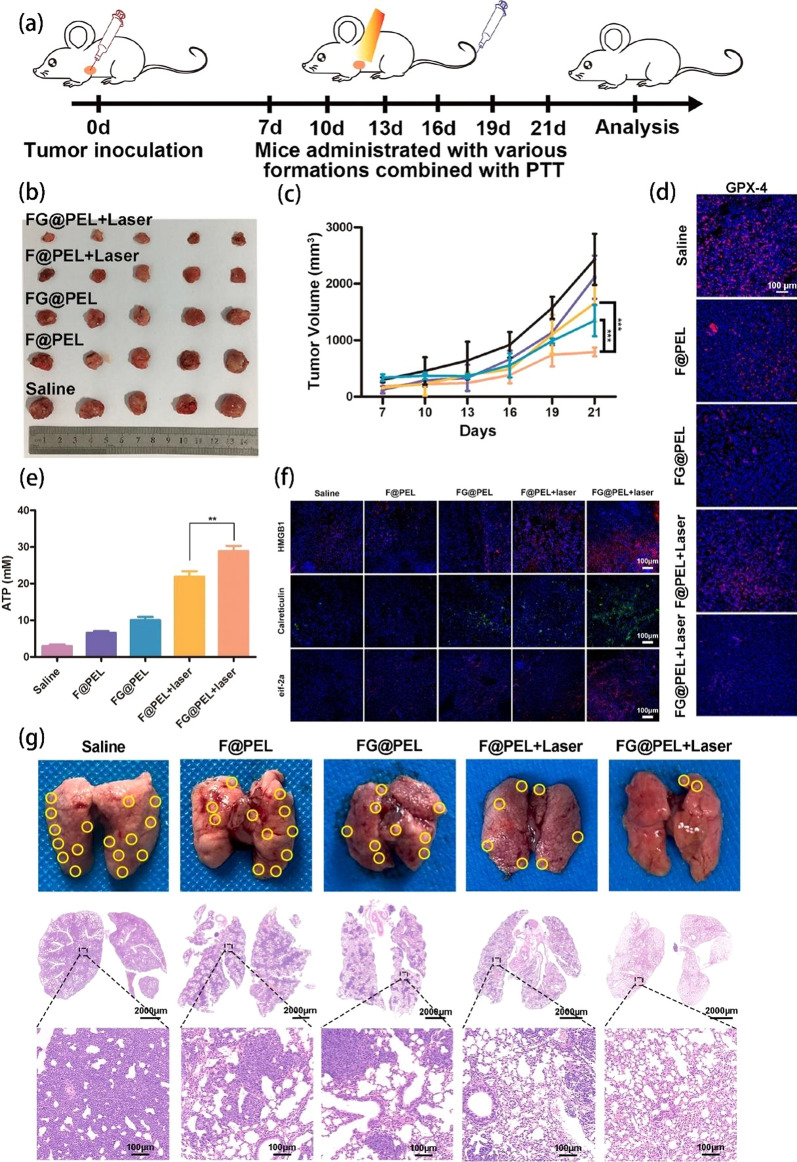
FG@PEL-based PTT for inhibiting tumor progression and lung metastasis in a 4T1 primary tumor mouse model in vivo. **a** Schematic diagram of FG@PEL-based PTT for inhibiting tumor progression in an H22 primary model. **b–c** Tumor volumes H22 tumor-bearing mice after different treatments. **d** Immunofluorescence assay examining the expression of GPX-4 in tumor tissues isolated from the aforementioned mice. Scale bar: 100 μm. **e–f** Levels of ATP, HMGB1, calreticulin, and eif-2a. **g** Lungs excised from 4T1 orthotopic tumor mouse models were used to evaluate the inhibitory effect of different treatments on pulmonary metastasis. H&E staining was performed on whole lungs isolated from these mice. Scale bar: 2000 μm. H&E staining of partial lung tissue. Scale bar: 100 μm. Reprinted with permission from Ref [184]

Phototherapy, which includes photothermal therapy (PTT) and photodynamic therapy (PDT), is a promising non-invasive strategy for cancer treatment [183]. In PTT, heat treatment is used to damage the microvasculature at the tumor site. Platelets target the damaged blood vessels as well as the tumors owing to their high surface expression of P-selectin, which recognizes CD44 receptors on the surface of cancer cells. Hence, Ma et al. combined exosomes derived from platelets with photothermal-sensitive liposomes and added glucose oxidase (GOx) and ferric ammonium (FAC) to create a laser-controlled nanoplatform called FG@PEL. In this system, GOx and FAC could enhance the Fenton response at the tumor site and damage tumor cells, while the photothermal effect resulted in both vascular damage to achieve cascade targeting effects and accelerated -OH production by increasing GOx activity through warming. In an in vivo experiment, mice were inoculated with mouse hepatocellular carcinoma cells (H22) on one side of their body, following which five treatments were administered (Fig. 7a). The most effective tumor growth inhibition was observed in the FG@PEL group (Fig. 7b–c). Subsequently, GPX-4, an indicator of ferroptotic death in tumor tissue, was detected. The reduced expression of GPX-4 in the FG@PEL + Laser group indicated that this combination induced ferroptotic death following light irradiation (Fig. 7d). It also significantly enhanced the release of immunogenic cell death (ICD)-related molecules, thus initiating an immune response to inhibit tumor development (Fig. 7e–f). Subsequently, a lung metastasis model was established. Fewer lung nodules were observed in the FG@PEL + Laser group (Fig. 7g). Finally, a bilateral tumor model was established, and treatment at the primary tumor site led to reduced tumor volume and a prolonged lifespan in the FG@PEL + Laser with anti-PD-1 treatment group [184]. Liu et al. introduced black phosphorus quantum dots (BPQDs) into exosomes (EXO) via electroporation to construct BPQDs@EXO nanospheres. The small BPQDs had a high photothermal conversion efficiency and good biocompatibility, and the EXO membrane could protect BPQDs from external oxygen and water, preventing their degradation in physiological fluids. Meanwhile, the homing effect of the EXO membrane allowed BPQDs@EXO to effectively accumulate within tumors. In in vivo experiments, BPQDs@EXO inhibited the growth of distant tumors in mouse models of bladder cancer more obviously after PTT, and no recurrence was detected [185].

Zhu et al. combined PDT with glutamine metabolic therapy and found that this multimodal therapy was successful in strongly inhibiting tumor growth [186]. Du et al. used CD47-functionalized exosomes loaded with a ferroptosis inducer (Erastin, Er) and a photosensitizer (Rose Bengal, RB) and performed PDT using 532 nm laser irradiation. They found that the Er/Rb@ExoCD47 (laser) group exhibited the best tumor suppression, highest levels of total ROS and lipid peroxidation, and low toxicity. Thus, they could serve as a useful strategy for the treatment of malignancies [187].

Phototherapy is a promising tool for cancer treatment, but some photothermal agents/materials and photosensitizers are prone to degradation and instability (e.g., BP). Hence, coating with EVs could help overcome these defects and achieve better therapeutic effects.

#### Gene therapy

**Fig. 8:**
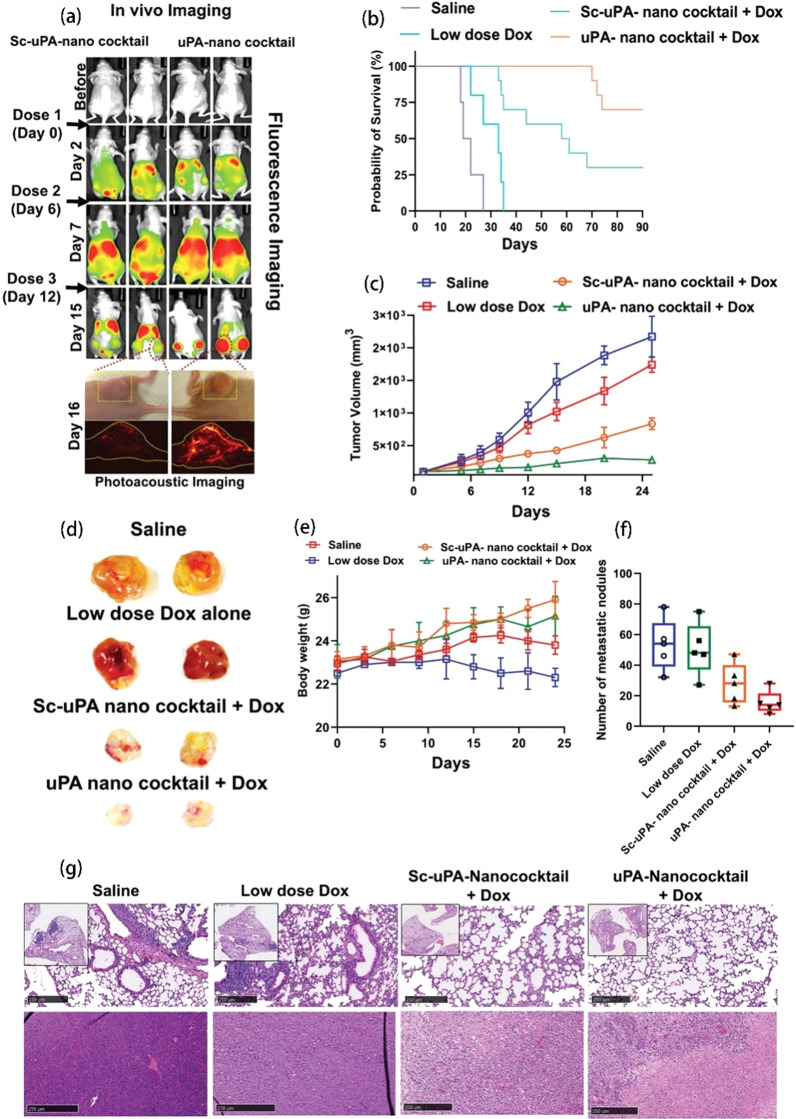
**a** In vivo fluorescence imaging showing the whole-body biodistribution and 4T1 tumor-specific accumulation of ICG-labeled Sc-uPA and uPA nano-cocktail formulations administered via tail vein injection on days 0, 6, and 12, and imaged on days 2, 7, and 15 using a Lago (Spectral Imaging system) in vivo imaging system. Photoacoustic imaging of 4T1 tumors to examine the accumulation of ICG-labeled eEV-uPA-PLGA-AntimiRNA on day 16. **b** Survival curves of 4T1 tumor-bearing mice treated with different formulations (Saline; Low-dose DOX alone; Sc-uPA nano-cocktail; and uPA nano-cocktail). Ten animals from each group were used for assessing the survival rate (n = 10). **c** 4T1 tumor growth kinetics following different treatments. **d** Ex vivo images of tumors excised 1 month after treatment. **e** Body weight of mice receiving different treatments. **f** Number of metastatic nodules in the lungs following different treatments. **g** Histologic assessment of mouse lungs and tumors using H&E staining. Reprinted with permission from Ref [188]

EVs intrinsically carry some RNAs and can thus be used directly for the treatment of some diseases. miRNAs are small non-coding endogenous molecules, and miRNA-10b and miRNA-21 were found to be overexpressed in TNBC. Therefore, Rajendran et al. used uPAR to modify EVs from tumor cells, thus increasing their natural tumor cell targeting ability. The EVs were loaded with polymeric nanocarriers (PNCs) containing anti-miRNA-10b and anti-miRNA-21 to generate a uPA-eEV-PNCs-AntimiRNA platform and exert combined antitumor effects. In vivo imaging showed that the fluorescence was initially clustered in the reticuloendothelial system, especially in the liver and spleen, However, it gradually clustered toward the tumor region after the administration of the second and third doses. The uPAR-targeted group showed greater fluorescence in tumors than the non-targeted SC-uPA group (negative control scrambled-uPA), and the photoacoustic imaging results revealed similar findings (Fig. 8a). uPA-eEV-PNCs-AntimiRNA was combined with low-dose DOX for inducing synergistic antitumor effects, and the survival time and survival rate observed after combined treatment were relatively good (Fig. 8b). The uPAR-targeted group showed stable inhibition of tumor growth (Fig. 8c–d), with negligible systemic toxicity (Fig. 8e) and reduced lung metastasis (Fig. 8f–g) [188]. Tao et al. loaded Bcl-2 siRNA into exosomes extracted from bovine milk using the ultrasonic method and demonstrated that the exosomes exerted strong anticancer activities both in vivo and in vitro [189]. Yuan et al. co-cultured HuCMSC-derived exosomes expressing miR-148B-3p with MDA-MB-231 cells and found that the exosomes inhibited cell growth. These exosomes were also demonstrated to inhibit tumor growth in vivo [190].

Nucleic acid-based gene therapy is currently gaining popularity in the field of cancer treatment. However, some RNAs have short half-lives and are prone to degradation, necessitating the development of nucleic acid vectors. In contrast, EVs carry nucleic acid substances within them, and can deliver RNA to target cells or tissues for therapeutic effects. Further, donor cells can be transfected to obtain the desired target RNA for treatment. Hence, gene therapy has good antitumor application prospects.

#### Combination therapy

**Fig. 9:**
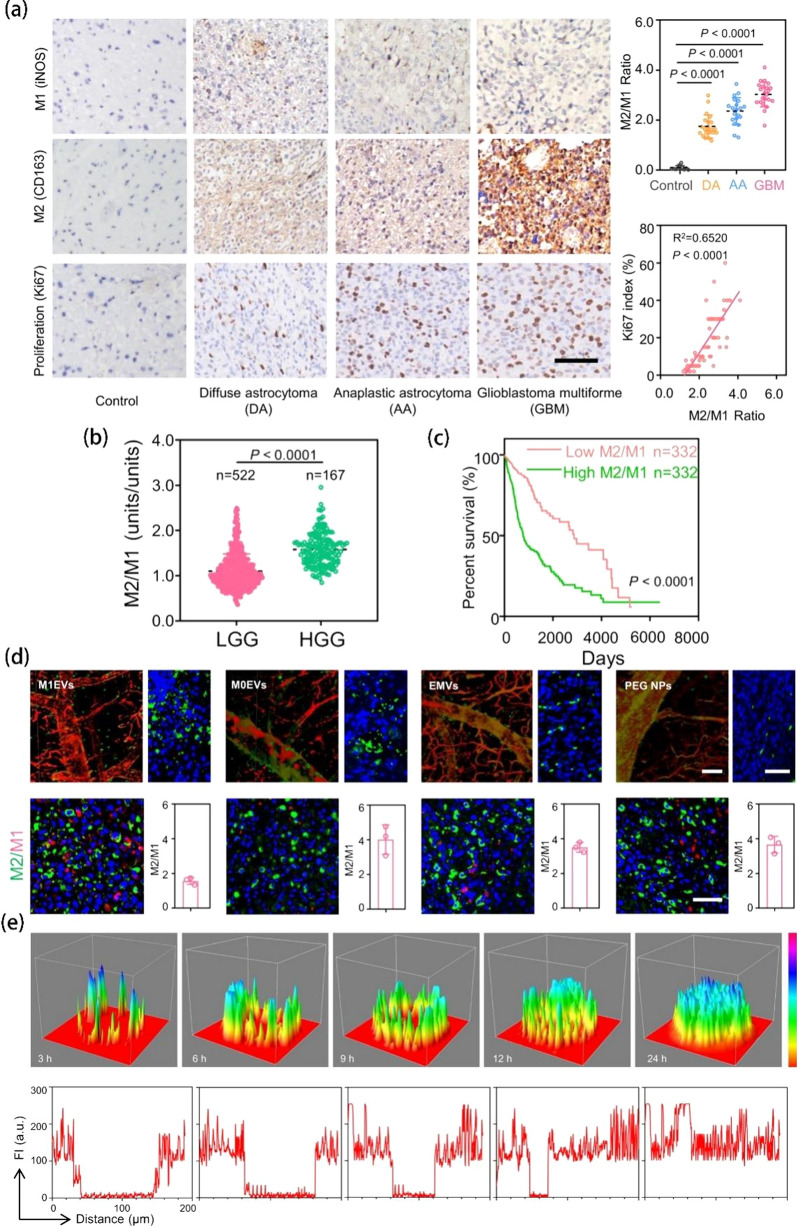
**a** Immunostaining of clinical histological sections obtained from normal tumor-adjacent tissues and low- (LGG: diffuse astrocytoma, n = 22) and high-grade gliomas (HGG: anaplastic astrocytoma, n = 20; glioblastoma multiforme, n = 22); M1 macrophages (iNOS), M2 macrophages (CD163), and cell proliferation (Ki67) are visualized. Quantitative analysis of the corresponding M2/M1 ratios is shown on the right side. The proliferation marker Ki67 was positively correlated with the M2/M1 ratio. Scale bars: 50 μm. **b** M2/M1 ratio analysis of 167 HGG and 522 LGG samples acquired from The Cancer Genome Atlas (TCGA) database. Each dot represents a single individual. **c** Survival curves of glioma patients from TCGA database. The OncoLnc tool was used to explore the correlations of survival with M2/M1 ratios. **d** In vivo time-lapse two-photon images of the diffusion of M1EVs, M0EVs, EMVs, and PEG NPs across microvascular endothelial cells of the brain at 48 h after i.v. injection (left). Tetramethyl-rhodamine isothiocyanate-Dextran was used to label blood vessels (red). M1EVs, M0EVs, and EMVs were labeled with DiO (green); PEG NPs were labeled with FITC (green); corresponding formulation distributions in tumor tissue are also shown (right). Scale bars: 50 μm. Immunofluorescence images of histological sections showing M2 (CD163, green) and M1 macrophages (iNOS, red) (left), and quantitative analysis of M2/M1 ratios (right) at 48 h after i.v. injection. Scale bars: 50 μm, (n = 3). **e** CLSM images and surface plots showing DiD-labeled M1EVs penetrating MCTS (top) and the corresponding fluorescence signal intensities across the spheroids (bottom). Reprinted with permission from Ref [191]

Monotherapy is often ineffective in killing tumor cells. Hence, researchers have adopted combination therapy for antitumor treatment. Glioblastoma (GBM) is a highly aggressive brain tumor. Through an analysis of clinical specimens, an increase in M2-like macrophages was observed in GBM patients. Therefore, Wang et al. proposed the use of M1-like macrophage-derived EVs (M1EVs) to modulate the tumor microenvironment (TME), and after aggregation at the tumor site, simultaneously polarize M2 into M1 and carry the chemotherapeutic drug bansoxadone (AQ4N) for post-release at the tumor site. The surface of EVs was modified with bis(2,4,5-trichloro-6-aminophenyl)oxalates (CPPO) and chlorin e6 (Ce6) to achieve synergistic effects of immunomodulation, chemically inspired photodynamic therapy, and hypoxia-triggered chemotherapy. A low M2/M1 ratio was positively correlated with low tumor proliferation and improved survival outcomes (Fig. 9a–c). The targeting effect of the EVs was confirmed using two-photon imaging, which revealed that M1EVs signals were abundantly present at the tumor site (Fig. 9d). The in vitro transwell co-culture system demonstrated that M1EVs loaded with different drugs could cross the BBB, and the integrity of the cell layer was confirmed using the tight junction marker protein ZO-1. The fluorescence intensity of DiD in the lower chamber was measured, and the penetration rate was found to be time-dependent, reaching approximately 30% at 8 h. The entry of M1EVs into multicellular tumor spheroids (MCTSs) improved with time after 24 h of incubation (Fig. 9e). Thus, macrophage-derived EVs showed great potential in the treatment of brain diseases [191]. Huang et al. designed lung-homing tumor-derived exosomes (Tex) hybridized with paclitaxel liposomes (Liposome-PTX) and incorporated PEG-gold nanorods (GNR) to achieve synergistic antitumor effects. Tex preferentially targeted the lung tissue, and GNR could treat tumors via thermal ablation after infrared (IR) irradiation, triggering both antitumor immune responses and PTX chemotherapy. Thus, this system showed potential as a clinical treatment option for advanced breast cancer recurrence and metastasis [192]. Pan et al. extracted exosomes from urine, achieving a high purity rate. They embedded synthetic nanoparticles within purified exosomes to inhibit tumor growth by blocking EGFR/AKT/NF-κB signaling via synergistic low-dose chemo-dynamic kinetics [193].

Due to the heterogeneity of tumors and the complex TME, researchers have shifted their focus from monotherapy to a combination therapy approach. The plasticity and load-bearing capacity of EVs enables the incorporation of multiple combination therapy modalities. Further, their inherent biocompatibility and targeting properties enable the delivery of multiple anti-cancer drugs or materials that can exert synergistic effects.

#### Others

**Fig. 10:**
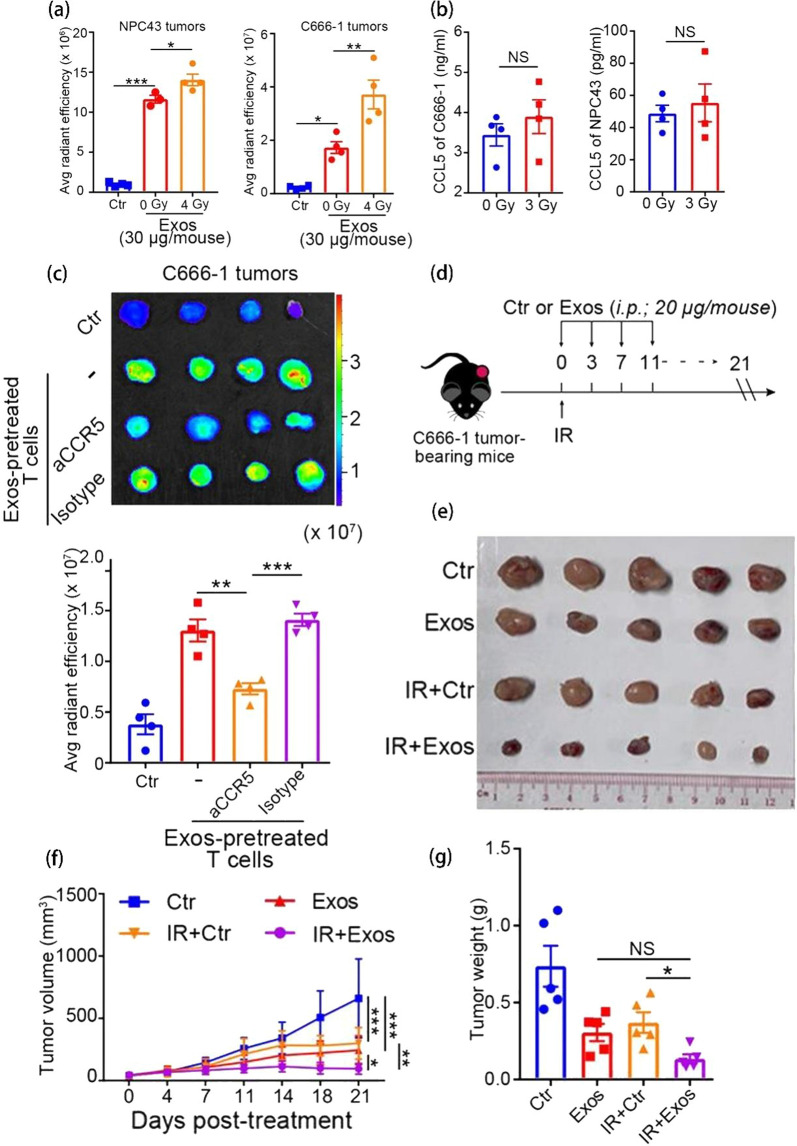
**a** Radiotherapy enhances the uptake of Exos that promote T cell migration into the NPC tumor microenvironment. After 24 h of treatment, the fluorescence intensity of CFSE in NPC cells was determined using flow cytometry. NPC43 or C666-1 tumor-bearing mice (n = 3 or 4) were irradiated (0 or 4 Gy) and treated with DiR-labeled Exos (30 µg/mouse) after 3 days. **b** CCL5 in the culture SNs of C666-1 and NPC43 cells 24 h after irradiation at 0 or 3 Gy. **c** Ex vivo detection (top) and analysis of DiR signals in tumor tissues (bottom). **d** C666-1 and NPC43 tumor-bearing Rag2^−/−^γc^−/−^mice (n = 5) were irradiated (0 or 4 Gy) and then intraperitoneally injected with Exos (20 µg/mouse) or Ctr. **e** Excised tumors, **f** Tumor volume, and **g** Tumor weight of C666-1 xenografts after treatment. Reprinted with permission from Ref [194]

Tumor necrosis factor-related apoptosis-inducing ligand (TRAIL) is a pro-apoptotic tumor factor. Ke et al. used the sensitizer dinaciclib (Dina) in combination with EVs secreted from TRAIL-carrying cells (EVs-T) to significantly enhance apoptosis in a variety of cancer cells. In vivo imaging showed that the delivered EVs-T were tumor- and organ-targeted and could effectively inhibit the development of drug-resistant tumors in vivo. γδ-T cells are lysis-active T cells that do not depend on the major histocompatibility complex and have inherent antiviral and antitumor activities [173]. Wang et al. used γδ-T cell-derived exosomes (γδ-T-Exos) in combination with radiotherapy to treat nasopharyngeal carcinoma. They found greater γδ-T-Exos accumulation at tumor sites in irradiated mice than in non-irradiated ones on in vivo imaging (Fig. 10a). Nasopharyngeal carcinoma cells secrete C-C chemokine ligand 5 (CCL5), which interacted with CCR5 and exerted a chemotactic effect on T cells. Irradiation did not alter CCL5 secretion (Fig. 10b), and human T cells could be recruited to nasopharyngeal tumors (Fig. 10c) through this nanosystem. Efficacy evaluation was performed (Fig. 10d) by comparing the effects of control treatment, irradiation, and single treatment on the inhibition of tumor growth (Fig. 10e–g) [194]. EGFR is highly expressed in GBM tissues and cell lines. Chen et al. added anti-EGFR monoclonal antibodies on the surface of EVs (mAb-EV), which became capable of targeting tumors inside the brain after crossing the BBB. They thereby delivered verrucarin A (Ver-A) for treating GBM. Nevertheless, the pharmacokinetics and pharmacodynamics of mAb-EV-Ver-A need to be thoroughly evaluated through preclinical studies [195].

In summary, EVs carry nucleic acids and can act as therapeutic agents by enhancing the expression of nucleic acids through transfection and other means, thereby exerting antitumor effects. However, the antitumor effects of single therapy can be somewhat limited. Hence, studies are increasingly adopting combination therapies, such as chemotherapy combined with phototherapy and immunotherapy combined with phototherapy. Hence, EVs are very promising agents for the treatment of cancer and have the potential to be translated into clinical studies.

### Neurodegenerative diseases

Neurodegenerative diseases are chronic disorders of the central nervous system and usually result from the intracellular accumulation of misfolded/aggregated mutant proteins. These abnormal protein aggregates impair mitochondrial function and induce oxidative stress, resulting in neuronal cell death. In turn, neuronal damage causes chronic inflammation and neurodegeneration [196, 197]. However, the BBB limits the accumulation and entry of drug molecules into the central nervous system. This precludes an effective concentration of drug molecules from reaching the brain tissue, compromising efficacy. Therefore, in the treatment of such diseases, drug delivery is challenging. There is an urgent need to develop drug delivery systems that can traverse the BBB [198].

Current efforts to improve drug delivery across the BBB are focused on enhancing drug entry into the brain and limiting drug loss [199]. Zhao et al. first used a strategy involving the intranasal injection of EVs loaded with the neurotrophic factor GDNF into transgenic Parkin Q311(X)A mice. They then evaluated motor function over a year after treatment and observed improved mobility, reduced neuroinflammation, and increased neuronal survival, without any systemic toxicity [200].

**Fig. 11:**
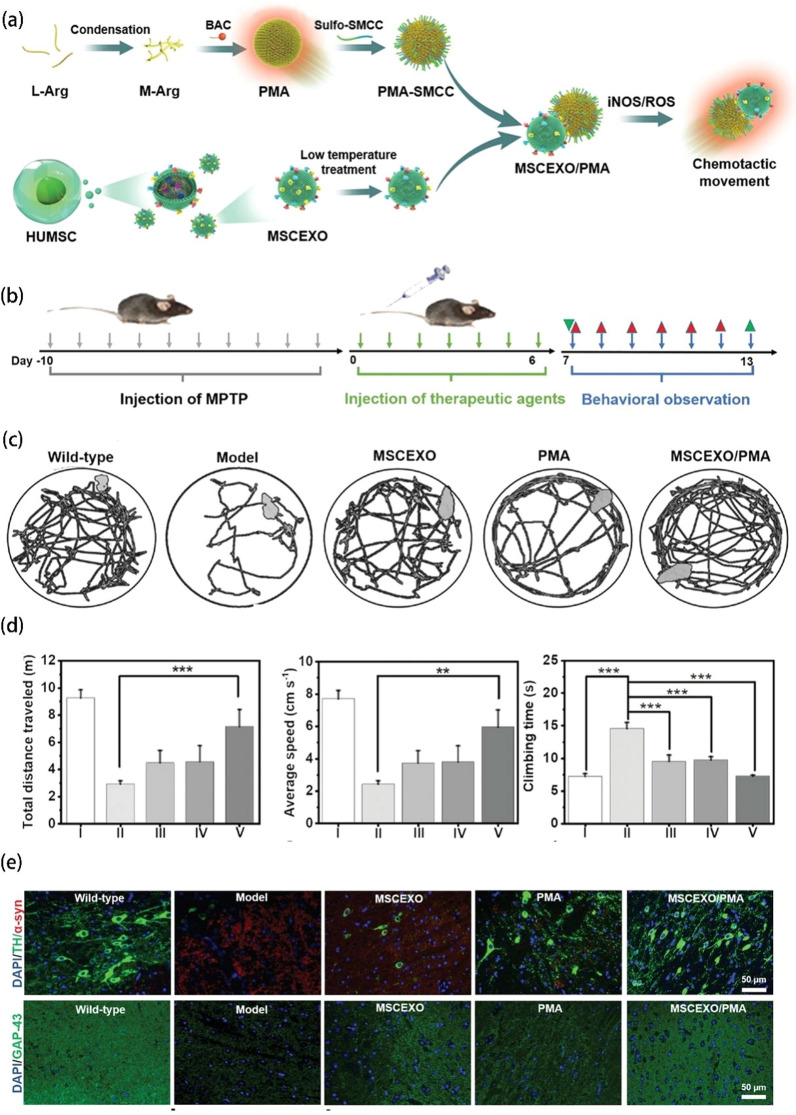
**a** Schematic illustration showing the construction of engineered exosomes with independent modules and their cascading function for the treatment of Parkinson’s disease (PD) via the multistep targeting and multistage intervention method. The blue line represents the action pathway of the natural exosome module MSCEXO, while the red line represents the action pathway of the artificial module PMA. **b** Drug treatment schedule, pathological monitoring, and therapeutic evaluation. Downwards green arrow: the open field and pole test; Upwards red arrow: Morris water maze training; Upwards green arrow: Morris water maze test. **c** Representative tracks showing mouse movement in the open-field test. **d** Total distance traveled and average speed of mice in the open-field test (n = 3), as well as the time taken to climb atop the pole in the pole test (n = 3). **e** Immunofluorescence staining: cell nucleus (DAPI, blue), TH (green), α-syn (red), and GAP-43 (green) (n = 3). Reprinted with permission from Ref [201]

Wang et al. proposed the concept of “independent module/cascade function”, in which nanoparticles with movement/chemotaxis abilities obtained from L-arginine acted as artificial modules that could bind to natural exosome modules (Fig. 11a). The guanidine group in arginine can react with iNOS and ROS in the PD microenvironment to generate nitric oxide (NO), providing motility to the engineered exosomes and increasing the degradation of α-synuclein (α-syn) aggregates, thereby promoting neuronal cell growth and enabling a functional disease treatment cascade. The effects of the strategy were examined in an MPTP-induced mouse model of PD (Fig. 11b) using the open-field test (parameters examined: walking trajectory, total walking distance, and average speed) and the pole test (parameters examined: time taken to climb atop the pole) (Fig. 11c–d). GAP-43, an indicator of neuronal growth, was found to be significantly upregulated in the substantia nigra (SN) region of the mouse brain. Further, α-syn aggregates in the SN were also found to be significantly downregulated (Fig. 11e). Based on these results, it appeared that the artificial module drives the natural module to cross the BBB and target damaged neuronal cells and mitochondria for the effective treatment of PD [201].

In contrast to exosomes produced by living cells, apoptotic vesicles produced by apoptotic cells are more useful. If manipulated, the apoptotic process can be controlled, and the vesicles can be loaded with drugs (such as nucleic acids) more efficiently than other EVs. Wang et al. first screened a subset of brain metastatic cells and eventually developed drug-loaded small apoptotic vesicles (Sabs) using melanoma cells. These vesicles carried anti-TNF-α antisense oligonucleotides. It was feasible to use Sabs as a novel EVs vector for drug delivery in vivo, especially for highly efficient siRNA or microRNA delivery, which is valuable for some in vivo biological applications [202]. Xue et al. proposed that MSC-derived exosomes can help in the treatment of PD by promoting ICAM1-associated angiogenesis. The presence of α-syn aggregates is a pathological feature of PD. Hence, α-syn downregulation is a potential strategy for PD treatment. While siRNA can achieve these effects, it has a short efficacy [203]. Izco et al. designed an shRNA microloop (shRNA-MCs) that can prolong the efficacy of siRNA and used RVG-exosomes as carriers for siRNA delivery to the brain, reducing the aggregation of α-syn and loss of dopaminergic neurons [204]. Hence, this system showed great potential in the treatment of neurodegenerative diseases. Kojima et al. developed exosomal transfer into cells (EXOtic) device that enables the efficient production of exosomes in engineered mammalian cells. The implantable exosome-producing cells could deliver therapeutic mRNA in vivo and reduce neuroinflammation and neurotoxicity in an in vitro model of PD [205]. Wang et al. used Cur to obtain Exo-Cur after ultracentrifugation following incubation with RAW264.7 macrophages. The addition of lymphocyte function-associated antigen 1 (LFA-1) on the surface of the exosomes enabled them to cross the BBB. The solubility and bioavailability of Cur were also enhanced. After targeted brain delivery, Cur could alleviate Alzheimer’s disease (AD) symptoms by activating AKT/GSK-3β and thereby inhibiting Tau protein phosphorylation [206]. Qi et al. also developed plasma exosomes carrying quercetin for the treatment of AD, improving both drug bioavailability and brain targeting [207]. This nanoformulation reduced cognitive dysfunction in mouse models of AD.

EVs are widely used in the treatment of neurodegenerative diseases because of their inherent ability to cross the BBB (due to the presence of surface proteins) and the advantages of better biocompatibility. However, the number of EVs that can actually reach the brain lesion site remains low. Hence, EVs must be modified to improve their targeting ability. How EVs interact with the BBB needs to be understood in more detail. Nevertheless, EVs represent an important platform for the future treatment of neurodegenerative diseases.

### Regeneration

Regenerative medicine refers to the restoration of the structure and function of damaged tissues via the repair of cells, tissues, and organs. Traditional stem cell therapy has safety issues, such as immune rejection and long-term survival challenges following systemic administration. In contrast, EVs have greater advantages in terms of biosafety, exogenous cargo delivery, and therapeutic effects [208].

**Fig. 12:**
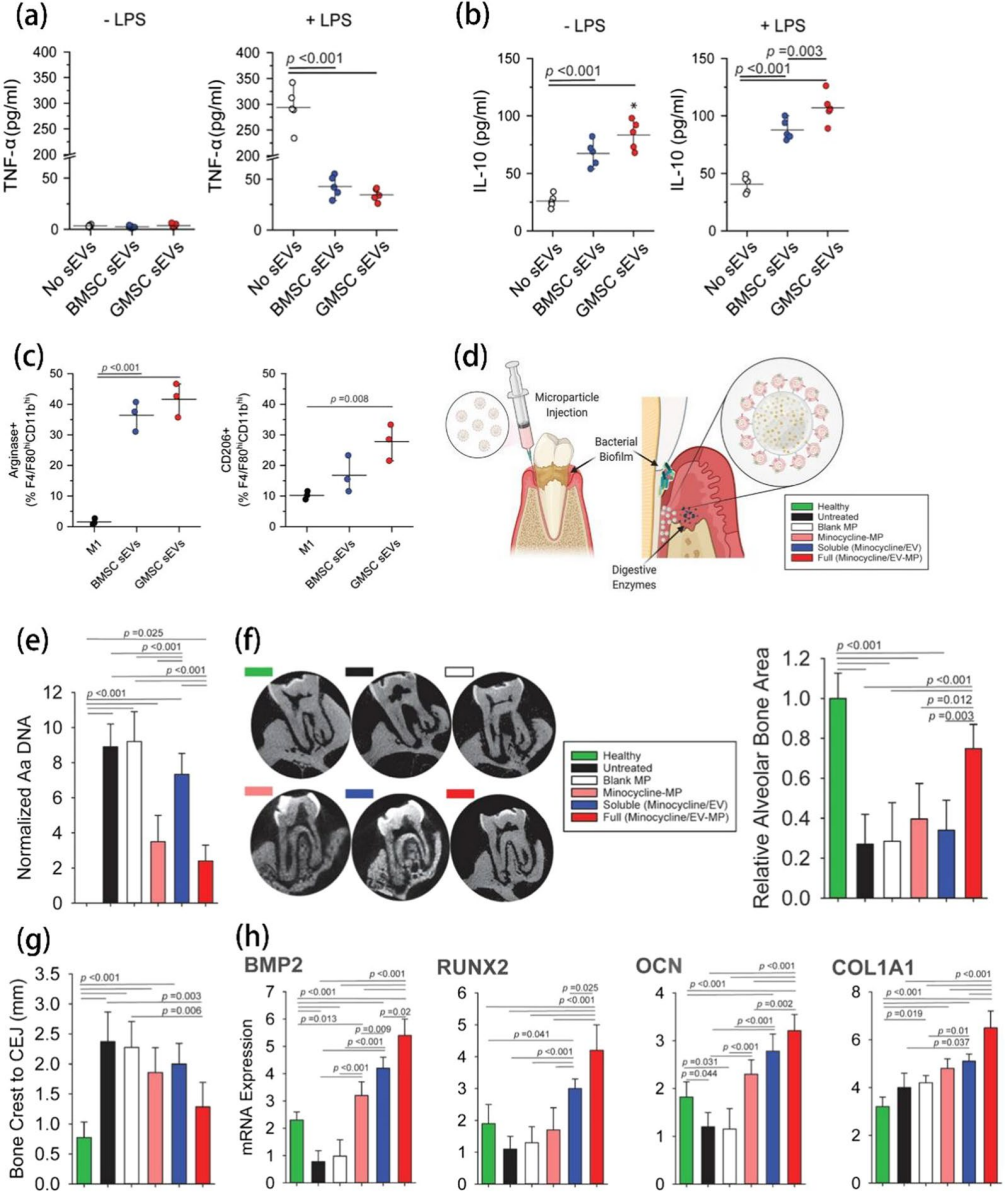
**a** Effect of sEVs on monocytes and macrophages. Change in the secretion of the inflammatory cytokine TNF-α after the addition of EVs to the monocyte culture. **b** Increase in the production of anti-inflammatory IL-10 by monocytes treated with sEVs, especially GMSC sEVs, despite the presence of LPS. **c** BMSC and GMSC sEVs stimulate macrophages to upregulate markers such as arginase and CD206, which are characteristic of the anti-inflammatory macrophage phenotype. **d–e** Schematics showing the administration of microparticles loaded with antibiotics and GMSC sEVs connected to the particles via an MMP-2-sensitive linker. Comparison of the effect of sEVs-microparticles on the suppression of bacterial growth evaluated 8 weeks after microparticle administration. **f** µCT images taken after 8 weeks of treatment and evaluation of the relative alveolar bone area in different treatment groups. **g)** Comparison of the distance between the alveolar bone crest and cementoenamel junction (CEJ). (**h)** Relative mRNA expression levels of BMP2, RUNX2, OCN, and COL1A1 in the periodontal tissue. Healthy—healthy rats; Untreated—untreated periodontitis; Blank—PLGA microparticles; Minocycline–MP—minocycline-loaded microparticles; Soluble—minocycline and GMSC sEVs administered in solution; Full—PLGA microparticles with GMSC sEVs and minocycline. Reprinted with permission from Ref [209]

EVs are currently used for the regeneration of bone, heart, lung, liver, kidney, and skin tissues. MSCs have great potential for immunomodulation and regeneration. Jana et al. extracted EVs from periodontal mesenchymal stem cells (GMSCs), applied them for periodontal tissue regeneration, and compared them with BMSC-EVs, which are commonly used for regeneration. Based on their respective effects on cytokine secretion and immune cell polarization, GMSC-sEVs (EVs sterilized by filtering through 0.22 μm filters with a particle size of around 100 nm) were found to enhance anti-inflammatory effects and decrease pro-inflammatory activity (Fig. 12a–c). Hence, GMSC-sEVs were selected for follow-up experiments. Particles containing antibiotics (minocycline) attached to sEVs were administered. The sEV-microspheres induced greater inhibition of bacterial growth (Fig. 12d–e). The amount of regenerated bone at the missing site was assessed using microcomputed tomography, and GMSC-sEVs produced a significant increase in bone area in the alveolus, similar to that in the healthy group (Fig. 12f). They also caused a significant decrease in the distance between the alveolar bone apex and enamel junction (Fig. 12g) and elevated the levels of osteogenic markers (Fig. 12h), indicating increased bone formation. The immobilization of EVs on the surface of PLGA particles via metalloproteinase-2 (MMP-2)-sensitive linkers, which could be cleaved by metalloproteinases present at the lesion site, allowed active drug localization at the lesion site and prolonged the retention time, thus promoting periodontal tissue regeneration [209].

Kim et al. extracted exosomes (Milk-exo) from bovine colostrum to treat alopecia. They observed hair regrowth on the backs of mice, with results comparable to those of minoxidil, indicating their great potential in the treatment of alopecia [210]. Xia et al. isolated exosomes from mouse wound edge fibroblasts and subsequently used them on mouse wounds. They found that myofibroblast abundance was increased during healing, and miR-125b transduced fibroblasts to inhibit sirtuin7 (SIRT7) and promote myofibroblast differentiation and wound healing in senescent mice [211]. Li et al. obtained MSC-BMP2-Exo liposomes incorporating the bone formation protein-2 (BMP2) gene, which was introduced into human fetal bone marrow MSCs. The modified exosomes promoted bone regeneration due to the synergistic effect of BMP2 upregulation and MSC-derived contents [212]. Lan et al. modified BMSC-derived EVs using neural EGFL-like protein 1 (NELL1), which stimulates bone formation, to enhance the bone repair process in vitro and in vivo. A 3D-Nell1/EVs-hydrogel was also found to promote bone regeneration in vivo, showing great potential in promoting bone healing [213]. Ko et al. used EVs for kidney regeneration and maintenance [214]. Meanwhile, Song et al. demonstrated that the multiple targeting of miR-210 by adipose stem cell-derived EVs could be valuable in the treatment of ischemic heart disease [215].

EVs themselves have functions such as the regulation of cell differentiation and promotion of angiogenesis, and they have similar biological properties as their source cells. This facilitates their wide application in regenerative medicine, including periodontal regeneration, osteogenesis, and tissue repair and regeneration in various organs and skin. Thus, they can serve as an alternative to stem cell therapy. However, several challenges need to be overcome before clinical application. These include the isolation and purification of EVs and the enhancement of their yield.

### Others

EVs have a wide range of therapeutic applications. In addition to the above-mentioned diseases, EVs can also be used to achieve anti-inflammatory effects and fibrosis inhibition. For example, Luo et al. found that BMSC-EVs significantly inhibit the fibrotic process and improve inflammatory phenomena and shoulder mobility by transducing let-7a-5p and inhibiting TGFBR1 [216]. Gao et al. proposed a method based on nitrogen cavitation to isolate EVs from cells, with a 16-fold higher yield and fewer subcellular organelles and genetic materials compared to naturally secreted EVs, and preserved cell membrane proteins. They found that the loading of anti-inflammatory drugs into EVs significantly attenuates lipopolysaccharide (LPS)-induced acute lung inflammation/injury and sepsis. The nitrogen cavitation method can be used to isolate EVs from any cell and produce EVs with a high yield, reproducibility, and scalability. These EVs can act as novel targeted delivery vehicles, and the increased yield could support clinical applications [217]. Yan et al. isolated exosomes from RAW 264.7 cells and loaded dexamethasone (Dex) into the exosomes using electroporation. The exosomes were surface modified with FA-PEG-Chol, which could actively target joint inflammation and reduce inflammation, improving the therapeutic effect of Dex on rheumatoid arthritis [218]. Ma et al. demonstrated that HuCMSC-EVs can act as bioactive agents to alleviate fibrosis in ligamentum flavum cells by delivering miR-146a-5p and miR-2213p, thereby inhibiting hypertrophy [219]. Han et al. obtained EVs from adipose-derived stem cells and used them in a thioacetamide-induced liver fibrosis model. They observed a significant reduction in collagen deposition and the restoration of liver function after treatment [220]. This model has also been applied to hypertension-related diseases. For example, Wang et al. reported the involvement of EVs secreted by endothelial cells damaged by hypertension via mechanical elevation in arterial wall remodeling, guiding renewed research into the treatment of diseases related to vascular wall remodeling [221]. Furthermore, EVs have also been used in the treatment of eye diseases. For example, Moisseiev et al. used saline and human MSC (hMSC)-derived exosomes to treat mice with oxygen-derived retinopathy (OIR) and found that the exosomes inhibited retinal thinning and alleviated retinal ischemia [222].

These findings indicate that good progress has been made in the application of EVs for achieving anti-inflammatory effects, fibrosis inhibition, and ocular management. By taking advantage of the natural properties of EVs, stem cell-derived EVs with anti-inflammatory and anti-fibrotic activities have been generated. These EVs could exert synergistic effects in combination with loaded anti-inflammatory drugs [223]. The value of EVs in the treatment of ocular diseases is also becoming more apparent.

In conclusion, EVs can serve as excellent nano-delivery vehicles or therapeutic drugs in their own right, and can be effective against a variety of diseases through the use of different therapeutic approaches. As delivery vehicles, EVs have relevant antigenic surface molecules that allow lesion targeting and BBB permeability, making them a novel therapeutic tool for the treatment of neurodegenerative diseases. In order to achieve better efficacy, different therapeutic tools can be combined, or EVs can be modified to obtain other functions. Thus, EVs show great therapeutic promise.

## Clinical trials of extracellular vesicles in COVID-19

In 2019, a new severe acute respiratory syndrome caused by severe acute respiratory syndrome coronavirus type 2 (SARS-CoV-2) emerged. This disease, known as coronavirus disease 2019 (COVID-19), was declared a pandemic by the World Health Organization (WHO) in March 2020. Since then, COVID-19 has attracted global attention. With a series of reports on SARS-CoV-2 variants, the situation remains unpredictable [224]. In particular, for resistant strains like Omicron, neutralizing antibodies and vaccines are less effective, and there is a lack of suitable drugs. However, researchers have found that exosomes can be used to transport anti-inflammatory cytokines that can effectively mitigate the effects of COVID-19 [225]. In contrast, MSCs with potent immunomodulatory activity have been proposed as treatment agents for COVID-19 because MSCs-derived exosomes activate M2 macrophage polarization to reduce inflammation. Further, they can increase the number and activity of neutrophils and reduce eosinophil and mast cell infiltration. These effects of exosomes can be attributed to the set of microRNAs (MiRs) with immunosuppressive and anti-inflammatory effects that they contain [226].

In 2020, 24 patients with severe COVID-19 received a single intravenous dose of 15 mL allogeneic BMSCs-derived exosomes at a hospital. Safety and efficacy were evaluated 1–14 days after treatment, and no adverse events were observed within 72 h of treatment. Of the 24 patients, 17 recovered, 3 remained in critical condition, and 4 died from non-treatment-related causes. However, clinical status and oxygenation improved after treatment, the cytokine storm was downregulated, and immune function was re-established [227]. In another clinical trial study, the safety and potential effectiveness of CD24-Exosome (EXO-CD24), a protein with anti-inflammatory properties, were evaluated in two tertiary care hospitals in Athens, Greece, in patients hospitalized with severe COVID-19. Patients received 10^9^ or 10^10^ exosome pellets per dose, once daily for 5 days, and were followed for 28 days. Notably, 72 of the 86 patients showed an improvement in respiratory rate and oxygen saturation. Also, 72 patients experienced at least a 50% reduction in inflammatory index levels from baseline (admission) by day 7. Further, no treatment-related adverse events were reported [228].

Exosomes have the advantage of being able to migrate to target organs instead of being captured by the lungs. Therefore, they have been approved for use as a therapeutic tool administered via nebulized inhalation [226]. Since, investigators reported the antiviral effect of recovering human immune plasma-activated exosomes (ChipEXO^™^) against SARS-CoV-2, where ChipEXO™ was a nebulized formulation that was passed through a jet nebulizer. The researchers administered this natural product to 13 COVID-19 patients with impending respiratory failure (1–5 × 10^10^ vesicles twice daily /5 ml distilled water for 5 days). After 5 days of treatment, ChipEXO™ was found to be well tolerated, causing no allergic reactions or acute toxicity. Elevent patients were cured, with no sequelae in the lungs and other organs, 2 h before and after exosome inhalation according to arterial blood gas analysis, indicating effective treatment. Oxygenation parameters and inflammatory markers were improved. Thus, ChipEXO™ showed good safety and efficacy [229].

In conclusion, COVID-19 remains a major concern in the world, and drugs are being tested for their treatment. With the rise of exosomes-based treatments, these strategies are being evaluated in clinical trials. For example, MSCs-derived exosomes are more widely used and can cause the apoptosis of activated T cells by inducing anti-inflammatory macrophages and regulating T and B cells. Thus, they are a potential therapeutic tool for the treatment of COVID-19. However, only a limited number of clinical trials based on MSCs have been completed. Hence, several challenges must be addressed for the application of EVs in COVID-19.

## Clinical challenges

While EVs hold great promise as therapeutic and drug delivery agents for disease treatment, and some EVs are already in clinical trials for disease treatment applications (clinical trials for EVs-based treatments are summarized in Table 6), but scalable production in compliance with regulations remains challenging [230]. Few EVs formulations are currently being translated clinically, and many problems need to be overcome before their universal clinical application.

**Table 6:** EVs-based therapeutics in clinical trials

Phase and number	Source/Sampling	Condition or disease	Dose or period of administration	Results or primary outcome measures	Refs.
Not Applicable n = 20	Human amniotic mesenchymal stem cells	Hair Loss; Alopecia	Exosome (100e10 particle) injections with an interval of 14 days during two months	Change in mean total hair density (hair/cm^2^)	NCT05658094
Phase 1 Phase2 n = 80	Human Placenta Mesenchymal Stem Cells	Fistula Perianal	In 3 weekly episodes	Safety of injected exosomes	NCT05402748
Phase 2 Phase3 n = 60	MSCs	SARS-CoV2 Infection	Intravenous injection twice, in day 1 and day 7 of 14 days	Time to clinical improvement (days)	NCT05216562
Phase 1 Phase2 n = 80	Human Placenta Mesenchymal Stem Cells	Perianal Fistula in Patients With Crohn’s Disease	5 mL of exosome solution	Safety of injected exosomes	NCT05499156
Phase 1 n = 24	Allogenic Adipose Mesenchymal Stem Cells	Coronavirus	5 times aerosol inhalation of MSCs-derived exosomes (2.0 × 10E8 nano vesicles/3 ml at Day 1, Day 2, Day 3, Day 4, Day 5)	Adverse reaction (AE) and severe adverse reaction (SAE)	NCT04276987
Phase 1 n = 30	Platelet rich plasma	Chronic Low Back Pain; Degenerativ-e Disc Disease	2 mL of exosomes	Visual analog scale (VAS)	NCT04849429
Phase 2 n = 30	MSCs	Knee; Injury; Meniscus (Lateral) (Medial)	1 million cells/kg Exosome	Evaluation of Knee Functions	NCT05261360
Not Applicable n = 30	N/A	Exosome Post-stroke Dementia; Acupunctur-e	N/A	concentration of Exosome	NCT05326724
Phase 1 Phase 2 n = 30	MSCs	COVID-19	Twice a day for 10 days inhalation of 3 mL special solution contained 0.5 ~ 2 × 10^10^ of nanoparticles (exosomes)	Number of Participants With Non-serious and Serious Adverse Events During Trial	NCT04491240
Phase 2 n = 41	Tumor Antigen-loaded Dendritic Cell	Non-Small Cell Lung Cancer	Intradermal injections once a week during 4 consecutive weeks	Progression free survival	NCT01159288
Early Phase 1 n = 9	Dendritic cells, macrophages, Tumor cells	Recurrent or Metastatic Bladder Cancer	N/A	Clinical response rate	NCT05559177
Phase 1 n = 13	Tumor cells	Malignant Glioma of Brain	10 to 20 million IGF-1R/AS ODN treated tumor cells, encapsulated in diffusion chambers (maximum of 10), and re-implanted in the patient’s abdomen within 24 h after the surgery for a 24-hour period	To establish the safety profile of a combination product with an optimized Good Manufacturing Practices AS ODN in the treatment of patients with recurrent malignant glioma with concomitant assessment of any therapeutic impact	NCT01550523
Phase 1 n = 38	Wharton’s Jelly Mesenchymal Stem Cells	Chronic Ulcer	Conditioned Medium gel for 2 weeks	Knowing the success rate of chronic ulcer healing in patients undergoing wound care with conditioned medium	NCT04134676
Phase 2 n = 102	Bone marrow	COVID-19, Acute respiratory distress syndrome (ARDS)	10 mL, which is 800 billion EVs. 15 mL, which is 1.2 trillion EVs	Evaluation of 60 day mortality rate	NCT04493242
Phase 1 n = 28	MSCs	Metastatic Pancreatic Adenocarci-noma ; Pancreatic Ductal Adenocarci-noma; Stage IV Pancreatic Cancer	over 15 ~ 20 min on days 1, 4, and 10. Treatment repeats every 14 days for up to 3 courses in the absence of disease progression or unacceptable toxicity. Participants who respond may continue 3 additional courses	Maximum Tolerated Dose Determined by Dose Limiting Toxicity	NCT03608631
Phase 1 Phase 2 n = 20	MSCs	Segmental; Fracture - Bone Loss	N/A	Adverse effects associated with the therapy	NCT05520125
Phase 1 Phase 2 n = 81	BMSCs	ARDS; Human	10 mL, 15 mL	The incidence of serious adverse events	NCT05127122
Not Applicable n = 25	Autologous blood	Otitis Media Chronic; Temporal Bone	N/A	Change in Inflammation Surface Area	NCT04281901
n = 300	Explore the source of extracellular vesicles	Traumatic Brain Injury	N/A	The type and content of circulating extracellular vesicles	NCT05279599
Phase 2 Phase 3 n = 100	Autologous blood	Otitis Media Chronic	N/A	Change of tympanic membrane perforation size	NCT04761562
Phase 1 n = 10	MSCs	Burns	1 × 10^4^ MSCs for each cm^2^	Primary Objective	NCT05078385
N/A	BMSCs	Covid19; ARDS; Hypoxia Cytokine Storm	Intravenous Infusion over 60 min	N/A	NCT04657458
Phase 1 Phase 2 n = 60	BMSCs	Covid19; Postviral Syndrome; Dyspnea	ExoFlo 15 mL (10.5 × 10^8^ EVs)	Increased distance on Six Minute Walk Test (6MWT)	NCT05116761
n = 50	Peripheral blood samples	ARDS Human	N/A	28 day mortality	NCT05061212
Not Applicable n = 10	Autologous serum	Ulcer Venous	3 weeks once a week	Changes in the ulcer area from baseline to eight weeks	NCT04652531
Phase 1 n = 10	Adult allogeneic bone marrow mesenchymal stem cell	Ulcerative Colitis	15mL of ExoFlo at Day 0, 2, 4 Week 2, 6, and every 8 weeks after that to week 46	Safety of intravenous ExoFlo in subjects with moderately to severely active Ulcerative colitis who have failed, or are intolerant, or have a contraindication to one or more monoclonal antibodies	NCT05176366
Phase 1 n = 10	Adult allogeneic bone marrow mesenchymal stem cell	Crohn Disease	15mL of ExoFlo at Day 0, 2, 4, Week 2, Week 6, and every 8 weeks after that to week 46	Safety of intravenous ExoFlo in subjects with moderately to severely active Crohn’s disease who have failed, or are intolerant, or have a contraindication to one or more monoclonal antibodies	NCT05130983
Phase 3 n = 400	BMSCs	COVID-19; ARDS	15mL, which is approximately 1.2 trillion EVs	The primary efficacy endpoint is overall 60 day mortality (due to any cause)	NCT05354141
Not Applicable n = 5	Adipose tissue	Wounds and Injuries	N/A	Percentage of wound healing in each group at 4 weeks	NCT05475418
Early Phase 1 n = 20	BMSCs	Solid Organ Transplant Rejection	Intravenous Infusion over 60 min	Number of participants with adverse/serious adverse events	NCT05215288
Early Phase 1 n = 10	Adipose stem cells	Periodontiti-s	N/A	change in gingival inflammation	NCT04270006
Not Applicable n = 30	MSCs	Foot; Diabetic	N/A	Ulcer evaluation	NCT05243368
Phase 1 n = 13	Autologous dendritic cell	Non-small cell lung cancer	Weekly, Four weeks	Survival of patients after the first DEX dose was 52 ~ 665 days. DTH reactivity against MAGE peptides was detected in 3/9 patients. Immune responses were detected in patients as follows: MAGE-specific T cell responses in 1/3, increased NK lytic activity in 2/4	[259]
Phase 1 n = 15	Autologous dendritic cell	Metastatic melanoma	Weekly, Four weeks	There was no grade II toxicity and the maximal tolerated dose, MAGE3 specific CD4 + and CD8 + T cell responses could not be detected in peripheral blood	[260]
Phase 1 n = 40	Ascites- derived exosomes (Aex)	Autologous Ascites	100, 200, 300, and 500 µg doses	the therapies were safe and well tolerated, the exosomes alone had no effect. After addition of colony stimulating factor, 1 case was stable and 1 case was mild	[261]
Phase 2 n = 22	Dendritic cell	Non-small cell lung cancer	Intradermal injections were given four times every other week interval	One patient exhibited a grade three hepatotoxicity. The median time to progression was 2.2 mo and median overall survival (OS) was 15 mo	[262]
Phase 1 n = 20	HL-60 cells	Biliary obstruction	20 mL MTX–TMPs containing 6 × 10^7^ tumour-cell-derived microparticles and 120 µg Methotrexate	Most patients (about 70%) had a transient fever (1–4 h) but no other uncomfortable symptoms and relieved biliary obstruction in 25% of the patients	[263]
Phase 2 n = 90	MSCs	SARS-CoV-2 PNEUMONIA	Twice a day during 10 days inhalation of 3 mL special solution contained 0.5 ~ 2 × 10^10^ of nanoparticles (exosomes)	Primary Outcome Measures: Number of participants with non-serious and serious adverse events during trial	NCT04602442
Phase 1 Phase 2 n = 30	MSCs	SARS-CoV-2 PNEUMON-IA	Twice a day during 10 days inhalation of 3 ml special solution contained 0.5 ~ 2 × 10^10^ of nanoparticles (exosomes)	Number of Participants With Non-serious and Serious Adverse Events During Trial	NCT04491240
n = 24	Allogeneic bone marrow mesenchymal stem cells	COVID-19	Single 15mL intravenous dose of ExoFlo and were evaluated for both safety and efficacy from days 1 to 14 post-treatment	N/A	[227]
Phase 2 n = 90	MSCs	SARS-CoV-2 PNEUMON-IA	Twice a day during 10 days inhalation of 3 mL special solution contained 0.5 ~ 2 × 10^10^ of nanoparticles (exosomes)	Number of participants with non-serious and serious adverse events during trial	NCT04384445
Phase 1 n = 24	MSCs	Coronavirus	5 times aerosol inhalation of MSCs-derived exosomes (2.0 × 10E8 nano vesicles/3 ml at Day 1, Day 2, Day 3, Day 4, Day 5)	Adverse reaction (AE) and severe adverse reaction (SAE)	NCT04276987
Phase 1 n = 35	T-REx™-293 cells engineered to express CD24 at high levels	SARS-CoV-2	1 × 10^8^ ~ 1 × 10^10^ exosome particles per 2 mL saline	Primary safety endpoint: Adverse events	NCT04747574
Phase 1 Phase 2 n = 55	MSCs	Covid19; Novel Coronavirus Pneumonia; ARDS	2 × 10^9^, 4 × 10^9^, 8 × 10^9^ exosomes	Measure and report the number of participants with treatment-related-adverse events as assessed by CTCAE v4.0; for patients receiving ARDOXSO™, perinatal MSC-derived exosome therapy	NCT04798716

The main problem hindering clinical translation is the lack of quality control and standardization procedures [23]. Moreover, the environment, conditions, and parameters for the isolation and purification of EVs vary from laboratory to laboratory, which may affect their properties. For example, as mentioned before, the high centrifugal force applied in the ultracentrifugation method may cause mechanical damage to EVs or lead to contamination. Low EVs yield is also a hindrance to clinical translation. Hence, the number of cells of mammalian origin needs to be expanded to obtain more EVs. The culture conditions (cell passaging, density, and frequency of EVs collection) may affect yield and bioactivity, among other factors. Moreover, these culture conditions are now not limited to traditional culture dishes, but instead can be expanded to bioreactors [58]. However, methods for increasing the production of EVs during the 72 h latency period include starving cells (most common), increasing intracellular calcium levels, depleting nutrient-rich cultures, hypoxic conditions, heat stress, and the addition of exogenous agents such as calcium ion carriers. However, during this process, cells show asynchronous activity during the growth, stress, and cell death phases [230], which may affect some properties of EVs. In addition, the cellular origin of EVs is very important and needs to be further characterized and tested for clinical suitability [5].

When EVs are used as therapeutic agents or vectors, it is crucial to quantify them precisely in order to determine the effective dose for subsequent administration. Since EVs themselves contain proteins, lipids, and nucleic acids, they can be quantified based on their total protein content, total lipid abundance, total RNA content, particle number, and the presence of some specific molecules. One drawback of protein quantification using the BCA, Bradford, or fluorescence methods is the interference of protein contamination or aggregation occurring due to improper separation. Particle number can be assessed using light scattering techniques, scanning electron microscopy (SEM), atomic force microscopy (AFM), and other assays, but there are certain requirements for particle concentration and size. In total lipid quantification, there is a variation in different lipid components. Thus, not all EVs can be detected, and some EVs are not sensitive to lipid detection. While RNA quantification in EVs is highly distorted, non-EVs RNA is abundant [231]. Therefore, the quantification techniques for EVs should be improved to allow their clinical translation. Moreover, when EVs are used as a carrier, they are usually engineered to target the lesion site. It remains to be examined whether the new substances introduced in this process can adversely affect patients, cause off-target effects, or lead to poor drug loading rates and poor efficacy, among other issues.

Another challenge precluding the clinical translation of EVs is stability. After the large-scale isolation and purification of EVs, a suitable environment is needed for storage to ensure their stability. Initially, EVs are resuspended in PBS and then stored at −80 °C for a long period. However, this makes transportation more difficult and costly, and also affects the physical and biological activity of EVs [232]. The addition of alginose can improve exosome aggregation and ameliorate cryoinjury [233]. Therefore, simple and convenient storage methods can be developed, and storage containers can be selected to improve the stability and minimize the loss of EVs.

The most critical challenge associated with EVs is safety. It is crucial to understand whether EVs injection into the body can produce adverse effects. A few studies have mentioned safety issues such as immunogenicity, immunotoxicity, and carcinogenicity. For example, tumor-derived EVs appear to be a double-edged sword. At first, tumor-derived exosomes were thought to promote tumorigenesis, metastasis, and angiogenesis. However, in subsequent studies, tumor antigens and heat shock proteins on the surface of these EVs were found to stimulate immune responses against tumor cells, and the inhibitory molecules could reduce cytotoxicity and act as antitumor vaccines [234]. Balancing the two during clinical translation in antitumor therapies is imperative. During in vivo studies, the effective dose conversion from animal models to humans may not be accurate. Thus, ideally, animal models that are most representative of the human disease should be used to explore the true efficacy of the system and guide clinical translation.

In conclusion, before the clinical translation of EVs-based therapies, we must first standardize the technique of EVs separation and purification as much as possible. A more complete preparation method is required so as to guarantee the purity and integrity of extracted EVs. Meanwhile, quantitative standards need to be unified to facilitate subsequent dosing. Secondly, it is important to improve the yield of EVs and maintain the homogeneity of EVs during the extraction process while controlling costs. The third consideration is stability. Improper storage may alter the stability and biological activity of EVs. Thus, a suitable storage method must be chosen. The final and most important aspect is safety. Before formal application to clinical settings, the safety of EVs preparations needs to be tested repeatedly to avoid complications such as immunogenicity and toxicity.

## Conclusion and future perspective

With the rise of bionanotechnology, nanomedicines have gradually caught the attention of the general public. EVs have been widely studied due to their low immunogenicity and ideal biocompatibility. Their ability to act as therapeutic agents and brain-targeting carriers that cross the BBB, which solves the problems associated with cell membrane-based bionanotechnology, has attracted the interest of researchers. They have been explored as therapeutic options for the treatment of cancer and neurodegenerative diseases as well as for regeneration. Depending on the diseases, EVs can be purified and isolated from a suitable source. Different techniques can be combined during isolation to maintain the purity or maximize the yield of EVs. subsequently, EVs can be used as therapeutic or drug-delivery agents and can also be modified and functionalized to achieve better results.

To obtain intact and uniformly distributed EVs for research purposes, it is crucial to use an appropriate separation and purification method. The current gold standard in research involves the use of ultracentrifugation and commercial kits for EVs separation, but some shortcomings still persist. Ultracentrifugation is operationally simple but is time-consuming, provides low purity, and can cause structural destruction. Meanwhile, commercial kits are time-consuming, offer low purity, and are expensive. Devices can help overcome these limitations and are more suitable for clinical application. However, they can only handle a few samples simultaneously. Thus, although many microfluidic-based separation methods have been developed, EVs yields would need to be improved to truly apply these devices clinically. Further, these devices would need to be improved to achieve high throughput and high purity and analyze all types of samples, while ensuring simple operation and developing automation.

For EVs as drug carriers, loading methods are divided into two broad categories: cargo loading before EVs separation and cargo loading after EVs separation. Different loading methods provide different loading efficiencies and stabilities. Currently, for gene therapy, the transfection method is preferred. However, the use of transfection agents can lead to contamination, and cargo loading with this method is not easily controllable. While electroporation is the gold standard method, it can affect the integrity of the membrane. Similarly, extrusion methods can cause lipid overturning. Therefore, compared to pre-loading methods, post-loading methods where cargo is loaded after separation appear to be more controllable. However, irrespective of the loading method, a low loading efficiency, destruction of the membrane structure, and inactivation or degradation of the loaded cargo continue to create challenges. In subsequent studies, attempts should be made to eliminate these disadvantages as much as possible and to combine advantages and develop an optimal method for loading cargo.

Besides applications in liquid biopsies, the replacement of tissue biopsies, and pain management, EVs can also be used as therapeutic agents or carriers for diseases such as cancer and neurodegenerative diseases and for regeneration. The effectiveness of EVs has been demonstrated through ex vivo experiments and has been attributed partly to their low immunogenicity, desirable biocompatibility, natural BBB penetration, targeting capacity, and potential to promote tissue regeneration. Therefore, when conducting research, the appropriate EVs need to be selected according to the disease of interest. As explained previously, tumor-derived EVs can both promote tumor growth and act as effective antitumor vaccines. This is encouraging provided that the advantages of these EVs are properly utilized. EVs are also involved in onset, development, and repair processes during the course of brain diseases. Hence, several studies are using EVs for the treatment of brain diseases. Some degree of efficacy has been reported, indicating that EVs are promising agents for the treatment of brain diseases. However, the mechanism through which EVs cross the BBB is not fully understood. Hence, more in-depth studies are needed. Moreover, only a few EVs reach the diseased areas of the brain. This problem also warrants urgent redressal.

However, in order to achieve better efficacy, the functionalization of the EVs surface is needed to improve their targeting ability and other characteristics. Functionalization is currently achieved by various methods and requires the selection of suitable ligands to avoid charge alterations, which can reduce stability. If functionalization is achieved by chemical reactions, irrelevant impurities must be removed to ensure safety. We should pay attention to whether the functionalization process will affect the inherent functions of EVs and their integrity. If such concerns exist, other functions can be added to EVs via functionalization methods, creating a valuable potential for biomedical applications.

In conclusion, through continuous optimization and improvement and subsequent regulatory approval, EVs formulations obtained from different sources could be successfully applied to the treatment of one or more diseases. Hence, they could provide a great contribution to therapeutic tools in the future.

## Data Availability

Not applicable.

## Abbreviations

EVs: Extracellular vesicles
BBB: Blood–brain barrier
MSCs: Mesenchymal stem cells
ISEV: International Society for Extracellular Vesicles
ILVs: Intracellular vesicles
MVBs: Multivesicular bodies
nSMase2: Neutral sphingomyelinase 2
MVs: Microvesicles
ROCK: RHO-related protein kinases
ARF: ADP-ribose factor
ERK: Extracellular signal-regulated kinase
MLCK: Myosin light chain enzyme
PS: Phosphatidylserine
TME: Tumor microenvironment
UC: Ultracentrifugation
DGC: Density gradient centrifugation
SEC: Size exclusion chromatography
UF: Ultrafiltration
MWCOs: Molecular weight cut-offs
TFF: Tangential flow filtering
sEVs: Small EVs
AF4: Asymmetric flow-field flow fractionation
HDL: High-density lipoprotein
LDL: Lipoprotein particles
HMSCs: Human neural stem cells
PEG: Polyethylene glycol
HOMM: Horseshoe-shaped mouth mixer
PS: Phosphatidylserine
TZ: Tetrazine
RT-dPCR: Reverse-transcription digital PCR
IMHPs: Immunomagnetic hedgehog particles
DDI: DNA-directed immobilization
USMB: Ultrasound combined with microbubbles
CUR: Curcumin
ADMSCs: Adipose-derived mesenchymal stem cells
HEK293T: Human embryonic kidney 293T
PTX: Paclitaxel
dHL-60: Neutrophil-like differentiated human promyelocytic leukemia
NDEVs: Neutrophil-derived extracellular vesicles
MSC-EXO: MSC-derived exosome
CTX/TRAIL: Cabazitaxel/Tumor necrosis factor-related apoptosis-inducing ligand
ADSCs: Adipose-derived stem cells
CTG: Cell tracker green
BSA- FITC: Bovine serum albumin coupled with fluorescein isothiocyanate
HUVECs: Human umbilical vein endothelial cells
HA: Hyaluronic acid
CV: Carvedilol
MM-EVs: Modulated-M1 EVs
THP-1: Human monocytes
PMA: Phorbol 12-mrustate 13-acetate
A15-Exo: Exosomal A15
CHO-miR159: Cholesterol-modified mi159
LND: Lonidamine
TNBC: Triple-negative breast cancer
LECs: Lens epithelial cells
MNPs: Magnetic nanoparticles
PR-EXO: RVG29 Peptide and Penetratin-Modified Exosome
CXCR4: Chemokine receptor 4
HuCMSCs: Human umbilical cord mesenchymal stem cells
CTF1: Cardiotrophin-1
BMCSc: Bone MSCs
OS: Osteosarcoma
TDEVs: Tumor-derived EVs
HCC: Hepatocellular carcinoma
hGLV: Lipid membrane-fused nanovesicles
HEs: Hybrid exosomes
An2: Angiopep-2
SDF-1: Stem cell recruitment factor
PDA: Dopamine
OXA: Oxaliplatin
CTL: Cytotoxic T cell
TMZ: Temozolomide
DHT: Dihydrotanshinone
IBA1: Inflammatory factor 1
PTT: Photothermal therapy
GOx: Glucose oxidase
FAC: Ferric ammonium
ICD: Immunogenic cell death
EXO: Exosomes
BPQDs: Black phosphorus quantum dots
PNCs: Polymeric nanocarriers
GBM: Glioblastoma
IR: Infrared
CCL5: C-C chemokine ligand 5
SN: Substantia nigra
LFA-1: Lymphocyte function-associated antigen 1
AD: Alzheimer’s disease
MMP-2: Metalloproteinase-2
BMP2: Bone formation protein-2
LPS: Lipopolysaccharide
OIR: Oxygen-derived retinopathy
SARS-CoV-2: Syndrome coronavirus type 2
COVID-19: Coronavirus disease 2019
WHO: World Health Organization
SEM: Scanning electron microscopy
AFM: Atomic force microscopy
